# The role of age-related genes in idiopathic pulmonary fibrosis and molecular docking analysis of their drug targets

**DOI:** 10.3389/fimmu.2025.1697013

**Published:** 2026-01-05

**Authors:** Wei Zhang, Tingting Xia, Qian Zhang

**Affiliations:** 1 Department of Geriatric Medicine, The First People’s Hospital, The Third Affiliated Hospital of Zunyi Medical University, Zunyi, Guizhou, China; 2 Department of Geratology, Affiliated The Tenth Affiliated Hospital of Southern Medical University (Dongguan People’s Hospital), Dongguan, China; 3 Department of General Medical Ward, The First People’s Hospital, The Third Affiliated Hospital of Zunyi Medical University, Zunyi, Guizhou, China

**Keywords:** age-related differential genes, clusterin(CLU), idiopathic pulmonary fibrosis, inulin, lipocalin-2 (Lcn2), meclizine

## Abstract

**Background:**

Idiopathic pulmonary fibrosis (IPF), a relentlessly progressive lung disorder marked by unremitting extracellular matrix deposition, continues to challenge clinical management due to its enigmatic etiology. Emerging evidence positions biological aging as a critical orchestrator of fibrotic reprogramming, where senescent cell accumulation and dysregulated tissue repair converge to drive disease progression.

**Methods:**

Three independent IPF transcriptomic datasets (GSE24206, GSE53845, GSE68039) were retrieved from the Gene Expression Omnibus (GEO) database. Aging-related differentially expressed genes (DEGs) were identified through intersection analysis with established senescence-associated gene sets. Functional annotation was performed using Gene Ontology (GO) and Kyoto Encyclopedia of Genes and Genomes (KEGG) pathway analyses. Protein-protein interaction (PPI) networks were constructed via STRING database and visualized using Cytoscape to identify topological hub genes. Competing endogenous RNA (ceRNA) networks and transcription factor (TF)-gene regulatory relationships were subsequently established. The DSigDB database was employed for drug-gene interaction prediction, complemented by molecular docking validation. Experimental validation was conducted using the GSE10667 dataset and a bleomycin-induced murine pulmonary fibrosis mode.

**Results:**

Comparative transcriptomic analysis revealed 292 DEGs between IPF and control tissues, with 19 exhibiting significant aging-related characteristics. Network topology analysis identified ten hub genes, including *CLU* and *LCN2*, that occupied central positions in both ceRNA networks and TF regulatory circuits. Drug enrichment analysis nominated inulin and meclizine as promising candidates demonstrating stable binding conformations with *LCN2* and *CLU*, respectively. External validation confirmed significant upregulation of *CLU* and *LCN2* in GSE10667 dataset, consistent with murine model findings.

**Conclusion:**

Our integrative analysis reveals novel molecular connections between cellular senescence programs and fibrotic lung remodeling, positioning *CLU* and *LCN2* as pivotal regulators of age-associated pulmonary fibrosis. The identified drug candidates exhibit therapeutic potential through multi-target engagement mechanisms, providing a translational framework for developing senescence-modulating therapies in IPF.

## 1 Introduction

Idiopathic pulmonary fibrosis (IPF) represents a relentlessly progressive interstitial lung disease characterized by pathological extracellular matrix deposition, culminating in irreversible pulmonary architecture distortion and progressive respiratory failure (1). Epidemiologically, this age-related disorder exhibits striking predilection for individuals over 60 years, with a median survival of 3–5 years post-diagnosis, highlighting the critical role of accelerated biological aging processes in disease progression (2). The archetypal usual interstitial pneumonia (UIP) pattern, defined by temporal heterogeneity and fibroblastic foci, has become a diagnostic cornerstone through advances in high-resolution computed tomography (3). While current therapeutic strategies (antifibrotics pirfenidone and nintedanib) demonstrate modest efficacy in slowing forced vital capacity decline (reducing annual loss by ~50%), their inability to reverse established fibrosis or substantially prolong survival underscores an imperative for mechanism-driven therapeutic discovery (4, 5).

The pathobiology of IPF involves a complex interplay of repetitive alveolar epithelial injury, maladaptive repair mechanisms, and dysregulated fibroblast activation, occurring in front of a backdrop of genetic susceptibility and environmental exposures (6, 7). Emerging evidence implicates cellular senescence as a pivotal amplifier of fibrogenic processes, where senescent alveolar epithelial cells and fibroblasts perpetuate tissue injury through senescence-associated secretory phenotype (SASP) mediators including IL-6, TGF-β, and matrix metalloproteinases (8). This pro-fibrotic secretome not only drives myofibroblast differentiation but also reshapes the immune microenvironment through chemokine-mediated recruitment of monocytes and alternatively activated macrophages, establishing a self-sustaining fibro-inflammatory circuit (8–11). Understanding the molecular mechanisms that connect aging with IPF is essential for identifying potential therapeutic targets.

**Figure 1:**
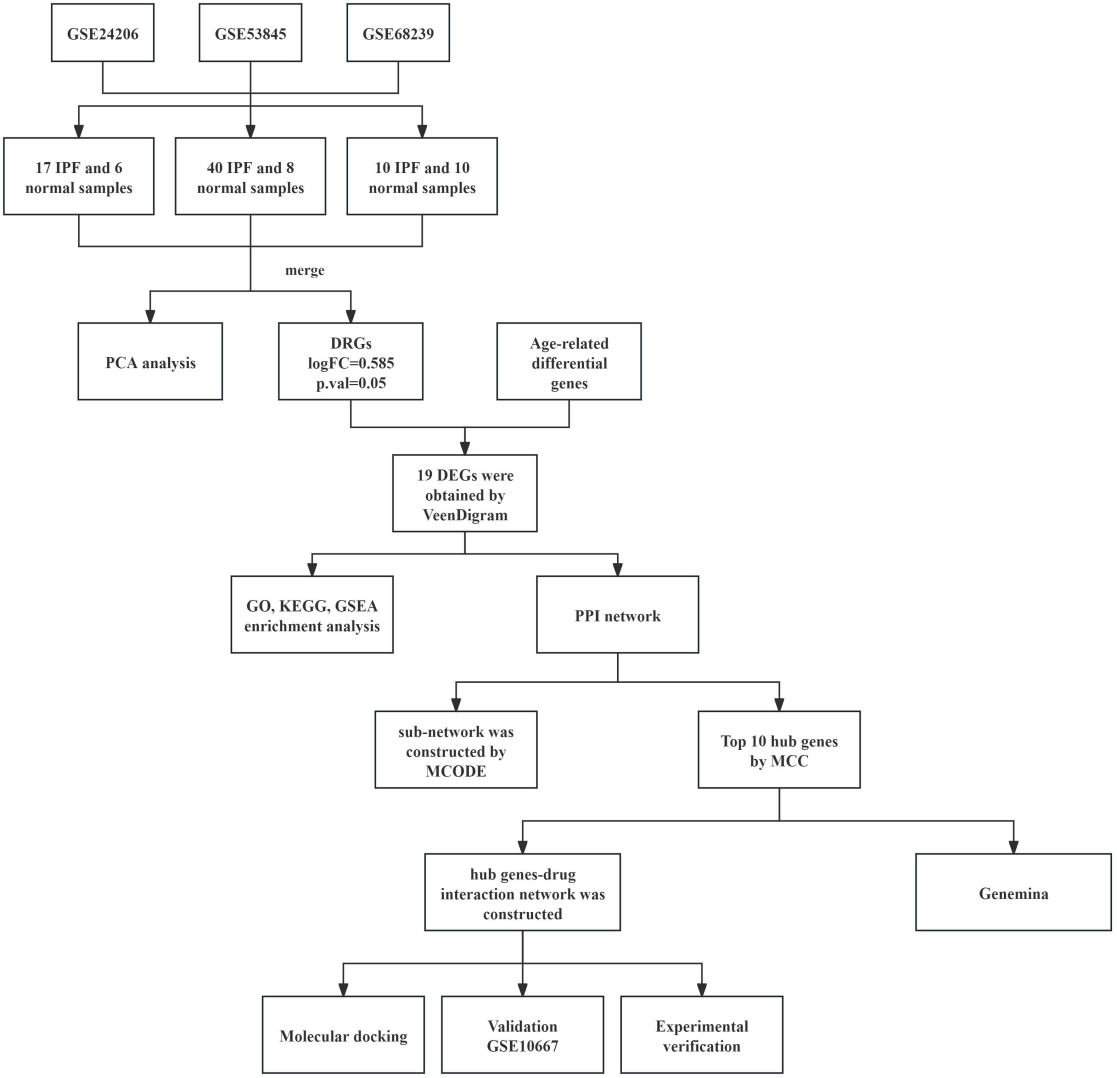
A flowchart depicting the sequential procedures involved in bioinformatics analysis.

In summary, this study utilized an integrated multi-omics approach to investigate the role of aging-related genes in the pathogenesis of IPF. Key senescence-associated genes, including CLU and LCN2, were identified and validated as significantly upregulated in both IPF patient samples and bleomycin-induced murine models. Furthermore, through computational drug screening and molecular docking analysis, inulin and meclizine were identified as potential therapeutic candidates for IPF (**Figure 1**). These findings provide novel insights into the molecular mechanisms linking cellular senescence to pulmonary fibrosis, offering promising avenues for the development of targeted anti-aging therapies in IPF.

## 2 Materials and methods

### 2.1 Data information

Transcriptomic discovery cohorts for IPF were systematically curated from the GEO database. Dataset selection was guided by predefined criteria to ensure robustness and generalizability: (1) studies must directly compare IPF lung tissue with healthy control samples; (2) inclusion of data from major gene expression profiling platforms (Affymetrix and Agilent) to enhance cross-platform consistency; and (3) sufficient sample size to support reliable differential expression analysis. Based on these criteria, three independent datasets were included: GSE24206 (17 IPF vs. 6 controls) generated on the Affymetrix HG-U133_Plus_2 platform (GPL570), GSE53845 (40 IPF vs. 8 controls) using the Agilent-014850 array (GPL6480), and GSE68239 (10 IPF vs. 10 controls) produced with the Agilent-012391 platform (GPL1708). For independent validation, the GSE10667 cohort (8 IPF vs. 15 controls), profiled on the Agilent-014850 array (GPL4133), was selected. Detailed clinical information for each dataset is provided in **Table 1**. This validation cohort was chosen due to its representation of an independent patient population and its use of a technologically comparable Agilent platform, enabling a consistent and unbiased evaluation of the identified gene signatures.

**Table 1:** Clinical information.

Features	Feature Subcategory	GSE24206		GSE53845		GSE68239	
		Number	Percentage	Number	Percentage	Number	Percentage
Total		23	100	48	100	20	100
Age	<60	5	21.74%	None	0	None	0
	≥60	12	52.17%	None	0	None	0
	unknown	6	26.09%	None	0	None	0
gender	Female	5	21.74%	9	18.75%	None	0
	Male	12	52.17%	39	81.25%	None	0
	unknown	6	26.09%	None	0	None	0

### 2.2 Principal component analysis

Technical reproducibility across experimental batches was systematically evaluated through principal component analysis (PCA), employing a rigorous computational workflow implemented in R (v4.3.1). Dimensionality reduction visualization was performed using the ggplot2 (v3.4.2) and ggpubr (v0.6.0) packages, with variance-stabilized expression matrices subjected to singular value decomposition following log2 transformation and quantile normalization.

### 2.3 Differential expression genes analysis

Cross-platform integration of the GSE24206, GSE53845, and GSE68239 datasets was achieved through the limma (v3.58.1)-sva (v3.50.0) framework, identifying 311 differentially expressed genes (DEGs) (|logFC|>0.585, adjusted *p* < 0.05, **Supplementary Table S1**), which were visualized via hierarchical clustering (pheatmap v1.0.12) and multidimensional scaling (ggplot2 v3.4.2) in the integrated cohort.

### 2.4 Analysis of age-related genes

Systematic intersection analysis between age-related genes (curated from the CellAge database, https://genomics.senescence.info/cells/, **Supplementary Table S2**) and IPF-associated DEGs using the VennDiagram package (v1.7.3) identified 19 genes exhibiting dual senescence- fibrosis regulatory potential.

### 2.5 Functional enrichment analysis of age-related differential genes

GO and KEGG pathway enrichment analyses were systematically conducted on age-related DEGs using the clusterProfiler (v4.10.0), GOplot (v1.34.0), and circlize (v0.4.15) R packages, with strict Benjamini-Hochberg multiple testing correction (FDR < 0.05) applied to ensure analytical rigor.

### 2.6 PPI network construction and hub gene identification

PPI networks of age-related DEGs were constructed using the STRING database (https://string-db.org/) with high-confidence interaction scores (>0.7) (12), followed by network visualization in Cytoscape (v3.9.1). Topological analysis was performed using the MCODE plugin (v2.0.0) to identify densely connected sub-networks, while maximal clique centrality (MCC) scoring via CytoHubba (v0.1) revealed the top 10 hub genes. Functional associations of hub genes were further validated through GeneMANIA (https://genemania.org/) using default multi-omics integration parameters.

### 2.7 Pharmacogenomic profiling of hub genes

A hub gene-drug interaction network was systematically constructed using the DSigDB database (https://dsigdb.tanlab.org/) to identify therapeutic compounds targeting IPF-associated hub genes. Pharmacological enrichment patterns were visualized through ggplot2 (v3.4.2) and annotated using org.Hs.eg.db (v3.18.0).

### 2.8 Molecular docking validation

Canonical 2D/3D drug structures were acquired from PubChem (https://pubchem.ncbi.nlm.nih.gov/) in SDF format, while protein tertiary structures were retrieved from the Protein Data Bank (http://www.rcsb.org/) with resolution thresholds (<2.5 Å) (13). Molecular docking simulations were performed using CB-Dock2 (https://cadd.labshare.cn/cb-dock2/) employing the hybrid scoring function (Vina Score + X-Score) with binding pocket detection sensitivity set to 0.85 (14).

### 2.9 Animal experiments

Healthy male C57BL/6 mice (6–8 weeks old) under specific pathogen-free (SPF) conditions were obtained from the Guangdong Medical Laboratory Animal Center (Guangdong, China) and maintained in a controlled environment (21–24 °C, 12-h light/12-h dark cycle). Following anesthesia via intraperitoneal pentobarbital sodium (40 mg/kg), the experimental cohort received intratracheal bleomycin (BLM, 5 mg/kg in 50 μL saline; Cat# B8416, Sigma-Aldrich) while controls received equivalent-volume sterile saline. Mice were euthanized under deep isoflurane anesthesia (5% induction) via cervical dislocation at 21 days post-induction, and tissues were immediately collected for histopathological analysis. All procedures strictly complied with arrive guidelines and were approved by the Experimental Animal Welfare and Ethics Committee of the Affiliated Hospital of Zunyi Medical University (Protocol# zyfy-an-2024-0706).

### 2.10 Histopathological evaluation and collagen quantification

The left lung was immobilized in 4% buffered formalin for 24 hours, dehydrated, embedded in paraffin, and cut into 4-micron sections for tissue staining. The histological changes of lung tissues in different groups of mice were observed through hematoxylin-eosin (HE) staining. Slices of mouse lungs were digitally imaged at 200× magnification (Olympus, Tokyo, Japan, VS200). Sections were stained using Masson’s trichrome staining according to the usual protocol to assess collagen deposition in the tissues.

### 2.11 Western blot

Total protein was isolated from the lungs of mice using RIPA lysis buffer supplemented with a protease/phosphatase inhibitors (15). Protein concentration was measured using the BCA protein assay kit (NCM Biotech, Suzhou, China). Proteins (20 to 30μg) were isolated using SDS-PAGE and transferred to PVDF membranes (Millipore, USA). Following a 1 hour blocking step with 5% non-fat milk at room temperature, the membranes were incubated with primary antibodies overnight at 4 °C. After three washes with TBST, the membranes were incubated with secondary antibodies (1:10,000, BA1038, BA1039, Boster, Wuhan, China) (16, 17) for 1 hour at room temperature. Proteins were then visualized using the Tanon Imaging System. The antibody details were as follows: anti-Clusterin (1:1,000, YP-mAb-00357, UpingBio, Hangzhou, China) and anti-LCN2 (1:1,000, YP-mAb-12477, UpingBio, Hangzhou, China).

### 2.12 Data analysis

All analyses were performed utilizing R software version 4.3.1 and Adobe Photoshop 2021. Data were presented as mean ± SD, and comparisons between groups were made utilizing an unpaired Student’s t-test. Statistical significance was defined as *p* < 0.05.

## 3 Results

### 3.1 Analysis of PCA and DEGs in IPF

**Figure 2:**
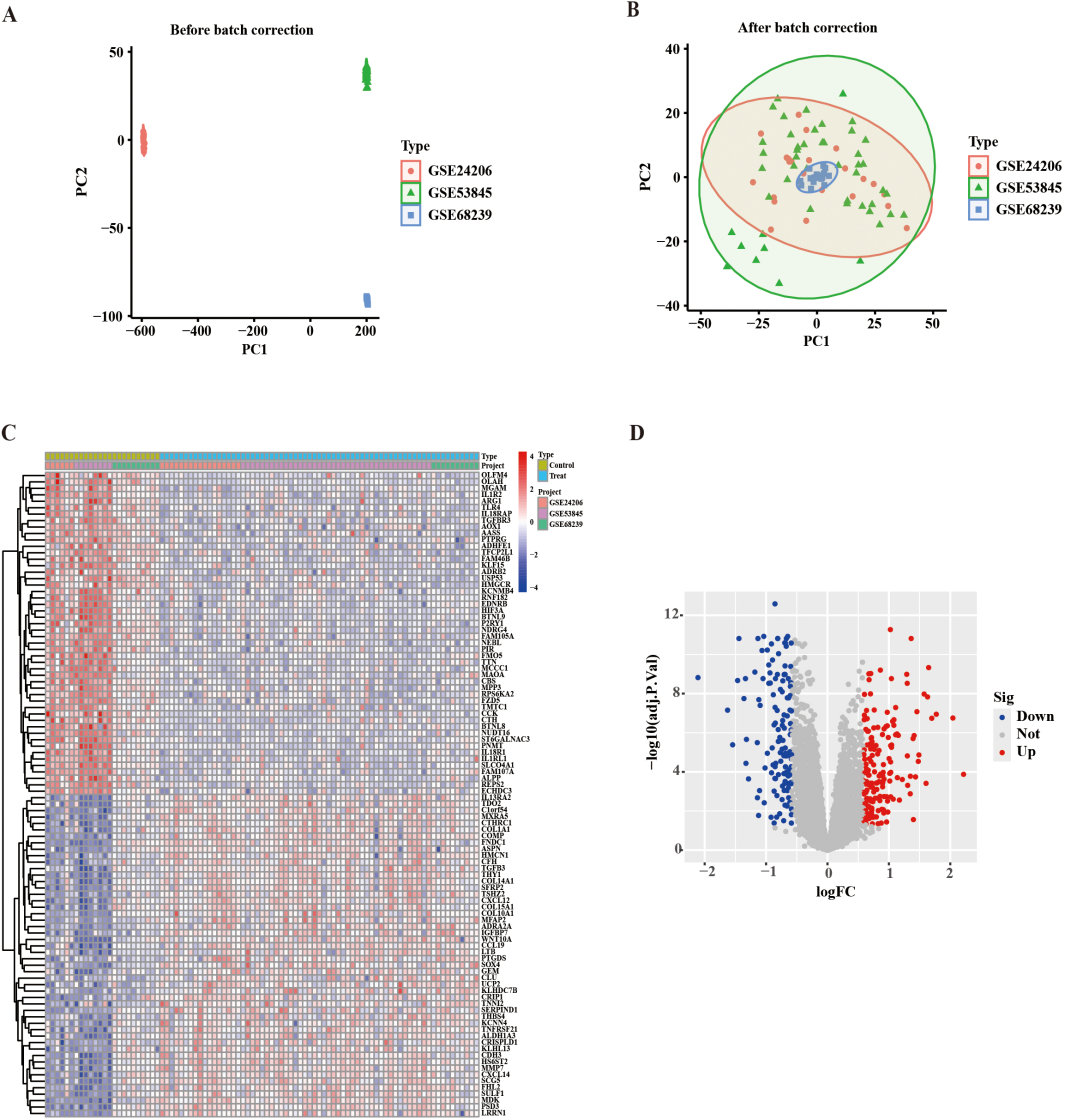
Identification of the DEGs in IPF. **(A)** PCA of transcriptomic profiles prior to batch effect correction. **(B)** The data after batch correction was analyzed by PCA. **(C)** A heatmap showing a total of 292 DEGs in the GSE24206, GSE53845, and GSE68239 datasets. **(D)** The volcano plot shows the expression of DEGs, with red indicating up-regulated genes and blue indicating down-regulated genes. DEGs, differential expression genes; PCA, principal component analysis.

Three independent IPF cohorts (GSE24206, GSE53845, GSE68239) were systematically analyzed through PCA after cross-platform batch effect correction, revealing distinct molecular clustering patterns between IPF and control samples (**Figures 2A, B**). Differential expression screening identified 292 DEGs (|log2FC| > 0.585, adjusted *p* < 0.05), including 176 upregulated and 116 downregulated transcripts. Heat map and volcano plot visualization demonstrated a robust separation of disease-associated transcriptional profiles (**Figures 2C, D**).

### 3.2 GO and KEGG enrichment analyses

**Figure 3:**
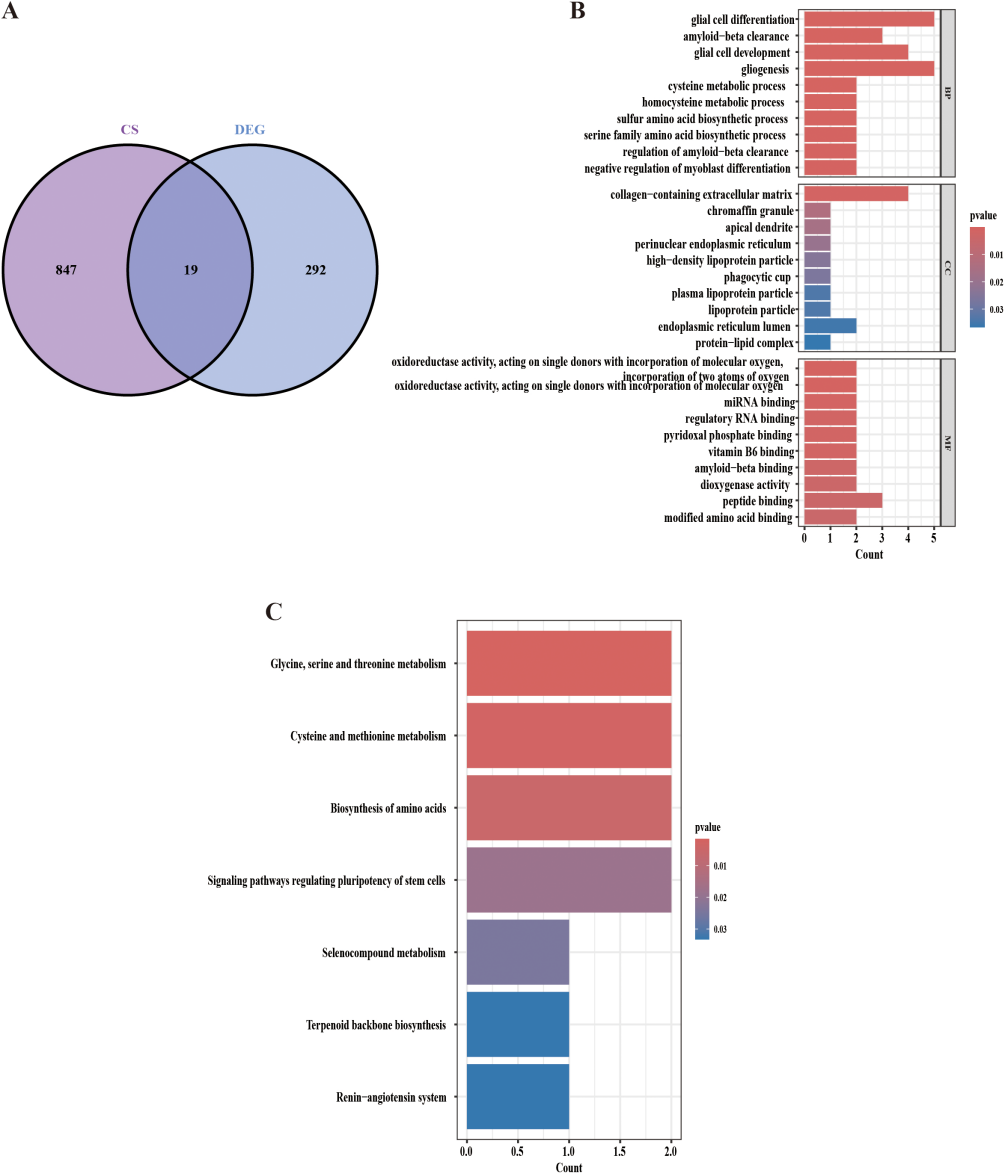
GO and KEGG enrichment analysis of aging-related differential genes. **(A)** Intersection analysis identifies 19 aging-related differential genes through Venn diagram visualization. **(B)** GO enrichment analysis of 19 aging-related differentially expressed genes. **(C)** KEGG pathway enrichment analysis of 19 aging-related differentially expressed genes. GO, Gene Ontology; KEGG, Kyoto Encyclopedia of Genes and Genomes.

To gain deeper insights into the functional relevance of aging-related genes in IPF, we intersected the 292 identified DEGs with a set of 847 aging-associated genes, identifying 19 overlapping aging-related differential genes (**Figure 3A**, **Supplementary Table S3**). Subsequent GO and KEGG enrichment analyses revealed several biologically meaningful pathways. Among the most relevant to IPF pathogenesis, these genes were significantly enriched in processes related to glial cell differentiation, amyloid-beta clearance, and extracellular matrix organization (**Figure 3B**, **Supplementary Figure S1A** and **Supplementary Table S4**). KEGG pathway analysis further highlighted key metabolic processes, including glycine, serine, and threonine metabolism, as well as cysteine and methionine metabolism pathways that have been previously implicated in fibrotic progression and cellular senescence (**Figures 3C**, **Supplementary Figure S1B** and **Supplementary Table S5**) (18, 19). These findings highlight key molecular mechanisms that may underlie the interplay between aging and IPF pathogenesis.

### 3.3 PPI network and core regulatory hub identification

**Figure 4:**
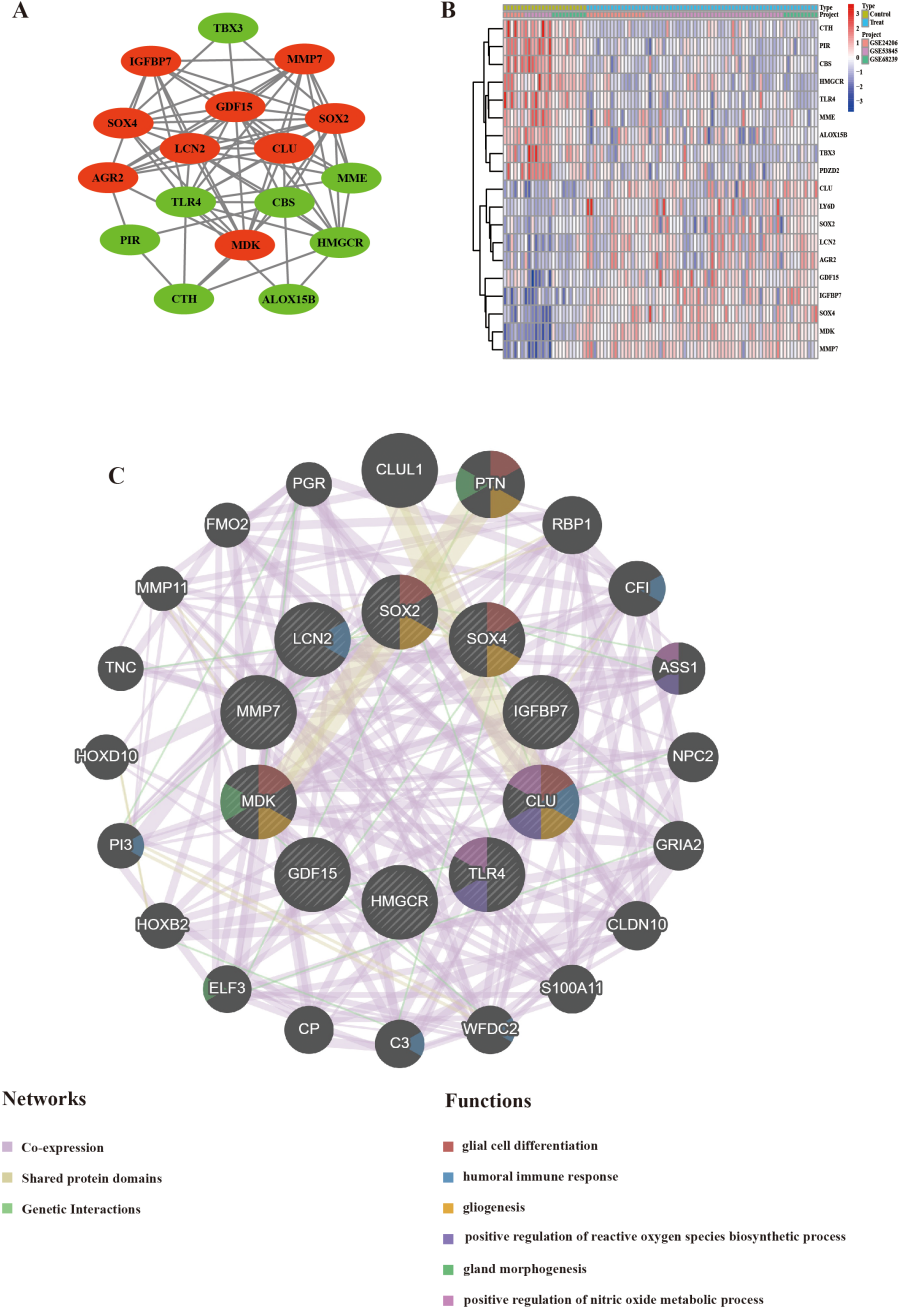
PPI network and hub genes of the aging-related differential genes. **(A)** Visualization of the PPI network. **(B)** Heatmap representation of expression patterns for the 17 differentially expressed genes in IPF patients compared to controls across three independent cohorts (GSE24206, GSE53845, and GSE68239). **(C)** PPI network of hub genes constructed using GeneMANIA. PPI, Protein-protein interaction.

A PPI network analysis of aging-related differentially expressed genes was performed using the STRING database, revealing functional interplay among the DEGs (**Supplementary Figure S2A**). Topological analysis using Cytoscape identified 17 differentially expressed genes, including nine upregulated (*AGR2, GDF15, MMP7, IGFBP7, CLU, SOX2, LCN2, MDK, SOX4*) and eight downregulated (*PIR, ALOX15B, HMGCR, TLR4, CBS, CTH, MME, TBX3*) genes within the expression-correlated interaction network (**Figure 4A**). Subsequently, heatmap analysis was performed to visualize the expression patterns of these 17 genes across multiple independent datasets comparing control and IPF subjects (**Figure 4B**). Furthermore, the MCODE algorithm extracted a high-confidence subnetwork, and the MCC scoring method was used to prioritize ten hub genes (*IGFBP7, CLU, HMGCR, SOX4, LCN2, TLR4, MDK, MMP7, GDF15, SOX2*) as core regulatory nodes (**Supplementary Figures S2B, C**). To further elucidate their functional interplay, a protein-protein interaction network was constructed for these ten hub genes using GeneMANIA, and a corresponding functional enrichment map was generated (**Figure 4C**).

### 3.4 Constructing the relationship between the hub gene and potential medications

**Figure 5:**
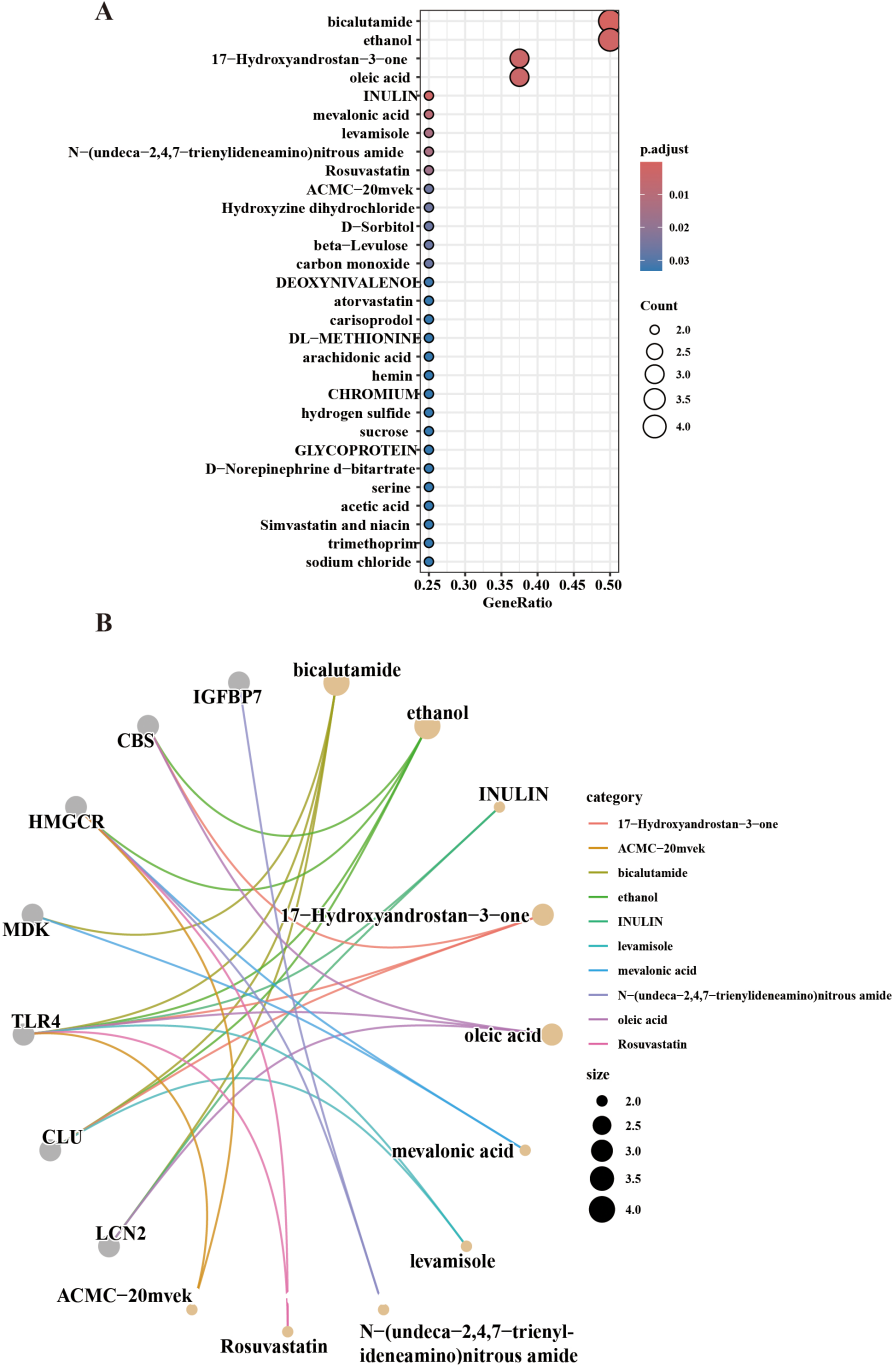
Drug enrichment analysis. **(A)** Bubble plot displaying the target genes enriched by the drug. **(B)** The top 10 most significantly enriched drugs associated with the target genes are shown.

The relationship data between hub genes and targeted drugs were obtained from the DSigDB online database, and a diagram illustrating the drug-gene binding was constructed (**Figure 5A**). The hub genes were found to be primarily associated with drugs such as bicalutamide, oleic acid, inulin, mevalonic acid, and rosuvastatin. These drugs may influence cellular senescence and exert anti-fibrotic effects in IPF (**Figure 5B**).

### 3.5 Forecast drug targets

**Figure 6:**
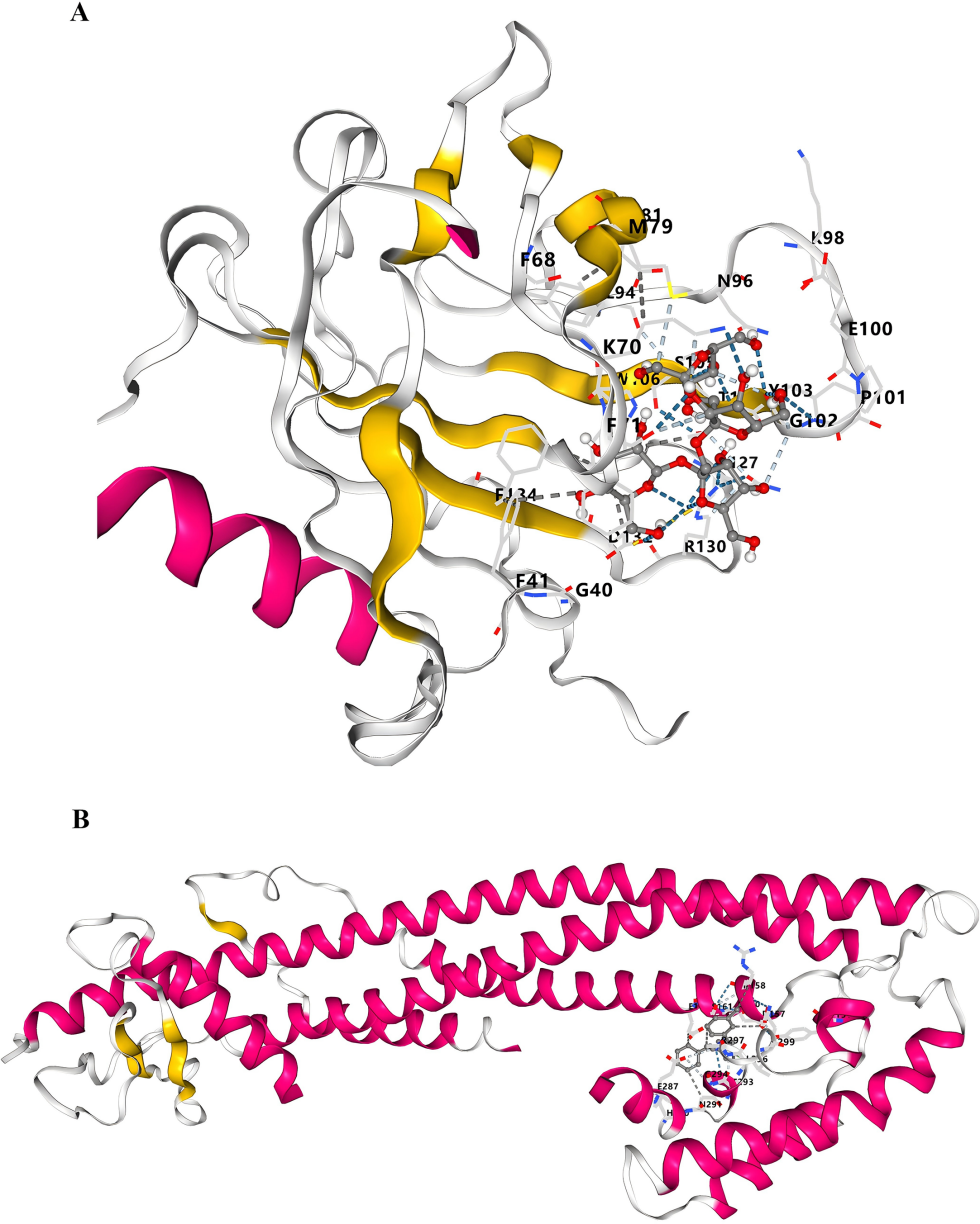
Molecular docking plots of inulin and meclizine with their respective proteins. **(A)** Molecular docking map of inulin with *LNC2*. **(B)** Molecular docking map of meclizine with *CLU*.

Drug enrichment analysis revealed that inulin and meclizine bind specifically to the LCN2 and CLU proteins, respectively (**Supplementary Table S8**). Although these compounds did not exhibit the most significant p-values in initial screening, they were prioritized for further validation based on their established anti-fibrotic efficacy in other organs (20–22). We hypothesize that through targeted modulation of LCN2 and CLU, these agents may attenuate pulmonary fibrosis progression, suggesting a novel therapeutic strategy for IPF. Subsequent molecular modeling integrated canonical 2D structural data from PubChem with tertiary protein structures from the PDB. Computational molecular docking simulations identified high-affinity binding pockets for both complexes. For inulin bound to *LCN2*, the top-ranking binding site (C1) exhibited a Vina score of -6.5 and a cavity volume of 1962 Å³, centered at coordinates (-12, 19, -21) with a docking grid size of (24, 24, 24) (**Figure 6A**, **Supplementary Table S9**). Another potential site, C5, also showed favorable binding (Vina score = -5.9) despite a smaller cavity volume of 63 Å³ (**Figure 6A**, **Supplementary Table S9**). For meclizine bound to *CLU*, molecular docking identified several high-affinity sites, notably C1 (Vina score = -7.7, cavity volume = 555 Å³) and C3 (Vina score = -7.2, cavity volume = 348 Å³), with detailed spatial parameters provided in **Figure 6B** and **Supplementary Table S10**. These results support the strong binding interactions and therapeutic potential of inulin and meclizine in IPF.

### 3.6 Validating the expression of CLU and LCN2

**Figure 7:**
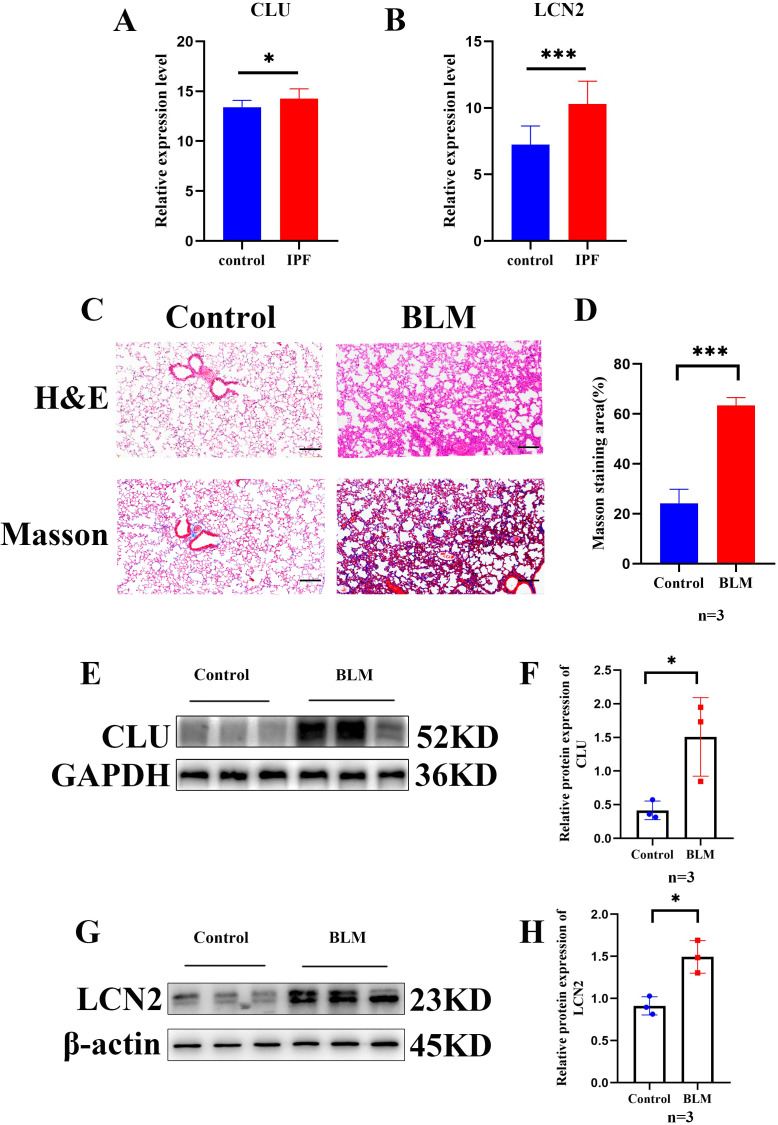
The expression of *CLU* and *LCN2* in IPF and control lung tissues from the online dataset and in murine model. **(A, B)** The expression of *CLU* and *LCN2* in the GSE10667 dataset. **(C, D)** H&E and Masson staining were conducted on mice lung tissue sections from the BLM group and the control group (original magnification ×200, scale bar: 100μm). **(E-H)** Western blot assays were employed to detect the expression of *CLU* and *LCN2* in mice lung tissues, n=3. *p < 0.05, ***p < 0.001.

We further validated the expression of the drug target genes *CLU* and *LCN2* using online datasets and an IPF mouse model. First, analysis of the GSE10667 dataset revealed that *CLU* and *LCN2* expression levels were elevated in IPF patients compared to healthy controls (**Figures 7A, B**). Next, pulmonary fibrosis was induced in mice via intratracheal instillation of BLM. Histopathological evaluation by H&E and Masson’s trichrome staining showed alveolar structural damage, thickened interlobular septa, and increased collagen deposition in BLM-treated mice relative to controls, confirming successful induction of pulmonary fibrosis (**Figures 7C, D**). Finally, gene expression analysis in mouse lung tissues demonstrated that both *CLU* and *LCN2* were upregulated in the BLM group (**Figures 7E-H**).

## 4 Discussion

Idiopathic pulmonary fibrosis (IPF) is a progressive and lethal interstitial lung disease characterized by dysregulated fibrogenesis and excessive deposition of extracellular matrix (ECM), ultimately resulting in respiratory failure and a five-year mortality rate exceeding 50% (23–27). The multifactorial pathogenesis of IPF involves a complex interplay between aging-related genomic instability, including telomere shortening and epigenetic modification, and exposure to environmental stressors such as particulate matter and microbial dysbiosis (28–30); however, the core mechanistic pathways underlying this interaction remain incompletely understood. Current therapeutic options for IPF are limited. Although antifibrotic agents like pirfenidone and nintedanib can reduce symptom burden and slow disease progression, their impact on long-term survival is modest (31–33). Given this unmet clinical need, the present study aimed to identify aging-related differentially expressed genes in IPF and to explore potential therapeutic compounds targeting these genes through molecular docking analysis, thereby offering novel insights into future therapeutic strategies.

Our integrated analysis of three independent cohorts delineated a distinct and robust transcriptomic landscape in IPF, defined by 292 differentially expressed genes (176 upregulated and 116 downregulated). These genes are functionally implicated in profound ECM remodelling and the induction of senescence-associated pathways. In particular, a cluster of upregulated genes, including *COL1A1, COL14A1, ASPN, CTHRC1* and *MMP7*, collectively fuels IPF progression by concurrently exacerbating ECM deposition and potentiating cellular senescence. Collectively, these alterations establish and sustain a pro-fibrotic microenvironment in the lungs of individuals with IPF, potentially through a self-reinforcing, senescence-dependent molecular circuit (34–40).

Cellular senescence, a pathophysiological state marked by irreversible cell-cycle arrest driven by telomere attrition, mitochondrial dysfunction, and genomic instability, emerges as a critical driver of IPF through SASP-mediated extracellular matrix remodeling (41–43). Nineteen overlapping genes were identified through the intersection of IPF associated DEGs and an aging-related gene set, including representative genes, which substantiates a direct molecular connection between pulmonary fibrosis and biological aging. GO enrichment analysis revealed that these genes are significantly involved in biological processes like glial cell differentiation. Multiple genes have been identified to play critical roles in glial cell function and pathology across different neural contexts. T-box transcription factor 3 (*TBX3*) is expressed in neural crest-derived glial cells, and its loss leads to reduced enteric glia without affecting neuronal density or gut motility (44). Lipocalin-2 (*LCN2*) regulates migration, morphology, and reactive astrogliosis in astrocytes and microglia via the Rho-ROCK pathway and upregulates GFAP expression under inflammatory conditions (45, 46). In the retina, SRY-box transcription factor 2 (*SOX2*) maintains Müller glia in a quiescent progenitor state, and its ablation results in morphological disruption and retinal degeneration (47). Clusterin (*CLU*) expression in Müller glia is modulated by inflammatory stress such as IL-1β, suggesting a role in photoreceptor suppor (48). Recent studies have revealed that glial cells (including astrocytes, microglia, and Müller cells) contribute to fibrotic processes in various organ systems through diverse mechanisms: mediating glial scar formation in the central nervous system (49), promoting fibronectin deposition via PAD4-dependent citrullination in the retina (50), driving fibrosis as “hepatic neuroglia” through norepinephrine -adrenergic receptor signaling in the liver (51), and participating in fibrotic microenvironment construction via Sox10+ cell expansion in the bone marrow (52). These evidences suggest that pulmonary fibrosis may involve similar neuroglial related mechanisms, such as sympathetic nerve activation regulating the activity of pulmonary fibroblasts through adrenergic receptors (53, 54), neuroglia-immune cell interaction mediated by the NF-κB pathway mediating chronic inflammatory responses (55), and tissue microenvironment remodeling induced by neuroglial- derived factors. These hypotheses await further verification in future experiments.

KEGG enrichment analysis revealed that these differentially expressed genes were significantly enriched in key metabolic pathways, including glycine, serine, and threonine metabolism, as well as cysteine and methionine metabolism. Emerging evidence has highlighted the role of sulfur containing amino acid metabolism in the pathogenesis of pulmonary fibrosis. For instance, STC1 has been shown to exert anti fibrotic effects by stimulating the cysteine glutathione pathway, which upregulates uncoupling protein 2 dependent demethylation of the SMAD7 promoter and promotes its acetylation (56). Meanwhile, methionine metabolism has also garnered attention: methionine synthase reductase (MTRR), a key enzyme in this pathway, has been identified as a potential therapeutic target (57). The drug clioquinol alleviates fibrosis by binding to MTRR, activating the methionine cycle, and increasing the production of methionine and S-adenosylmethionine, thereby suppressing fibroblast activation and extracellular matrix deposition (57). These findings collectively suggest that modulating cysteine and methionine metabolism may offer novel treatment strategies for pulmonary fibrosis through the regulation of cellular redox balance and epigenetic modifications. In contrast, direct evidence linking glycine, serine, and threonine metabolism to pulmonary fibrosis remains limited. Existing studies indicate that the metabolism of these three amino acids is often dysregulated in a coordinated manner in conditions such as metabolic liver disease, wherein supplementation strategies have been shown to ameliorate metabolic imbalance and reduce aberrant collagen synthesis, reflecting a broader role in tissue stress response (58–60). Thus, although direct mechanistic studies in the lung remain limited, it is plausible that dysregulation of these metabolic pathways may indirectly contribute to pulmonary fibrosis by disrupting collagen turnover and impairing tissue repair processes. This hypothesis, while conceptually compelling, requires rigorous experimental validation.

Our study systematically identified 10 hub genes (*IGFBP7, CLU, HMGCR, SOX4, LCN2, TLR4, MDK, MMP7, GDF15, and SOX2*) that act as dual-functional regulators of fibrotic remodeling and cellular senescence in IPF. Molecular docking analysis revealed high-affinity binding of the natural polysaccharide inulin to *LCN2* and the antihistamine meclizine to *CLU*, suggesting their potential to modulate these targets for anti-fibrotic therapy. *LCN2* plays multifaceted roles in IPF pathogenesis, acting as a biomarker of pulmonary inflammation, a modulator of oxidative stress, and a regulator of alveolar repair. Elevated *LCN2* expression in IPF lung tissues and bronchoalveolar lavage fluid (BALF) correlates with reduced respiratory function (61), positioning it as a surrogate biomarker of disease severity. In acute exacerbations (AE-IPF), elevated serum *LCN2* levels independently predict poor survival (62), while murine studies reveal its protective role against bleomycin-induced oxidative stress. *LCN2^−/−^* mice exhibit exacerbated lung injury and impaired antioxidant responses (62). Paradoxically, despite its association with neutrophilic inflammation in acute lung injury models, *LCN2* deletion does not mitigate fibrosis, suggesting its pathogenic role is secondary to inflammation-driven oxidative stress (61). Furthermore, *LCN2* overexpression suppresses alveolar epithelial type 2 (AT2) cell proliferation, a critical repair mechanism, though interleukin-17 (IL-17) partially rescues this deficit, highlighting the therapeutic potential of targeting the LCN2/IL-17 axis (63). *LCN2* may also play an important role in the progression of diseases such as Parkinson’s, breast cancer, and disc degeneration by regulating cellular senescence (64–66). *CLU* exhibits opposing functions in IPF depending on its subcellular localization. Extracellular *CLU* promotes alveolar epithelial apoptosis, whereas intracellular *CLU* enhances DNA repair and reduces senescence (67). IPF lungs exhibit a pathological shift toward extracellular *CLU* dominance, mirroring the fibrotic phenotype of *CLU^−/−^* mice, which display unresolved DNA damage and persistent fibrosis post-bleomycin injury (67). Transcriptomic analyses further link elevated *CLU* expression to extracellular matrix remodeling and disease progression (68), solidifying its dual roles as a driver of fibrogenesis and a guardian of epithelial homeostasis.

The anti-fibrotic potential of inulin and meclizine is supported by their established roles in mitigating fibrosis across organs. Inulin has been shown to significantly improve liver fibrosis (20) and effectively alleviate colon fibrosis caused by chronic radiation exposure (21), highlighting its therapeutic potential in managing fibrotic conditions across various organs. Similarly, in a mouse adenomyosis model, meclizine administration accelerated endometrial repair and improved endometrial fibrosis in a dose-dependent manner (22). We found that the expression of *CLU* and *LCN2* was significantly higher in IPF lung tissues than in controls using an external validation set and by constructing a mouse IPF model. Given the involvement of *CLU* and *LCN2* in the progression of pulmonary fibrosis and regulation of cellular senescence, it is hypothesized that inulin and meclizine may exert an anti-pulmonary fibrosis effect in the lungs by targeting these genes and modulating cellular senescence, offering a potential new therapeutic approach for IPF. While this study identifies promising therapeutic candidates, several limitations must be acknowledged: (1) The anti-fibrotic effects of inulin and meclizine in IPF remain experimentally unvalidated; (2) The relatively small sample size and the use of only a single time point in our animal experiments may introduce bias and limit the robustness of the findings; future studies with larger cohorts and multiple time points are warranted to strengthen our conclusions; (3) Clinical correlations between hub gene expression and patient outcomes are lacking; (4) Potential batch effects in transcriptomic data could introduce bias. Future work should prioritize *in vivo* validation of these compounds in IPF models, large-scale multicenter cohorts to confirm biomarker utility, and mechanistic studies to resolve CLU/LCN2’s compartment-specific roles.

Furthermore, it is noteworthy that the GSE68239 cohort displayed a distinct clustering pattern in the analysis compared to the other two datasets. This divergence is primarily attributed to technical batch effects inherent in integrating cross-platform microarray data, despite the application of standardized correction algorithms. Additionally, undefined biological heterogeneity across independent cohorts may further contribute to this separation. Importantly, this global-level technical variation does not undermine our core findings. The age-associated key genes, such as CLU and LCN2, identified through differential expression analysis, along with their enriched pathways, were consistently and robustly validated across all three integrated datasets. This consistency confirms that the central biological signals underlying our conclusions are strong and reproducible, effectively transcending the influence of technical artifacts.

## 5 Conclusion

In conclusion, our research provides significant insights into the molecular mechanisms underlying IPF by highlighting the role of age-related DEGs and their potential regulatory networks. Through the integration of transcriptomic data and bioinformatics analysis, we have uncovered complex interactions between these genes, identifying promising candidates for therapeutic targets and biomarkers. Furthermore, our findings indicate that inulin and meclizine may slow the progression of pulmonary fibrosis by targeting specific genes, offering potential avenues for treatment. This study lays a foundation for future research to translate these discoveries into effective clinical interventions, contributing to the advancement of IPF management and therapy.

## Data Availability

The datasets presented in this study can be found in online repositories. The names of the repository/repositories and accession number(s) can be found in the article/**Supplementary Material**.
