# Supplementary material for: The role of age-related genes in idiopathic pulmonary fibrosis and molecular docking analysis of their drug targets
Source: Front Immunol. 2026 Jan 5;16:1697013. doi: 10.3389/fimmu.2025.1697013 (PMC12812732; doi:10.3389/fimmu.2025.1697013)
Supplement: Supplementary file 1 [file DataSheet1.docx]

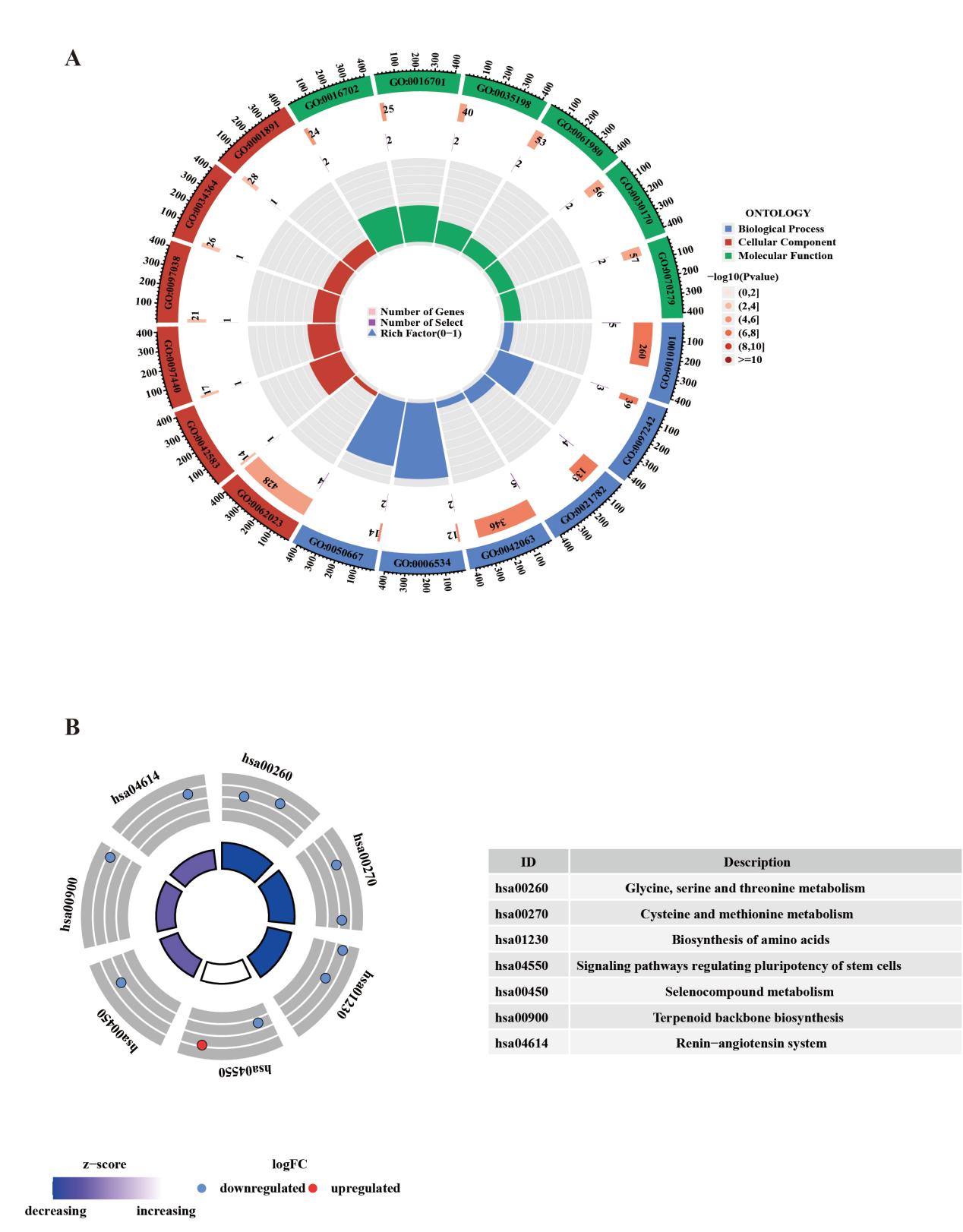


**Supplementary Figure 1** **GO and KEGG enrichment analysis**. **(A)** GO analysis delineates biological processes, cellular components, and molecular functions. **(B)** KEGG pathway enrichment analysis identified seven key pathways, which were prioritized by normalized enrichment score (NES) and validated through permutation testing. GO, Gene Ontology; KEGG, Kyoto Encyclopedia of Genes and Genomes.


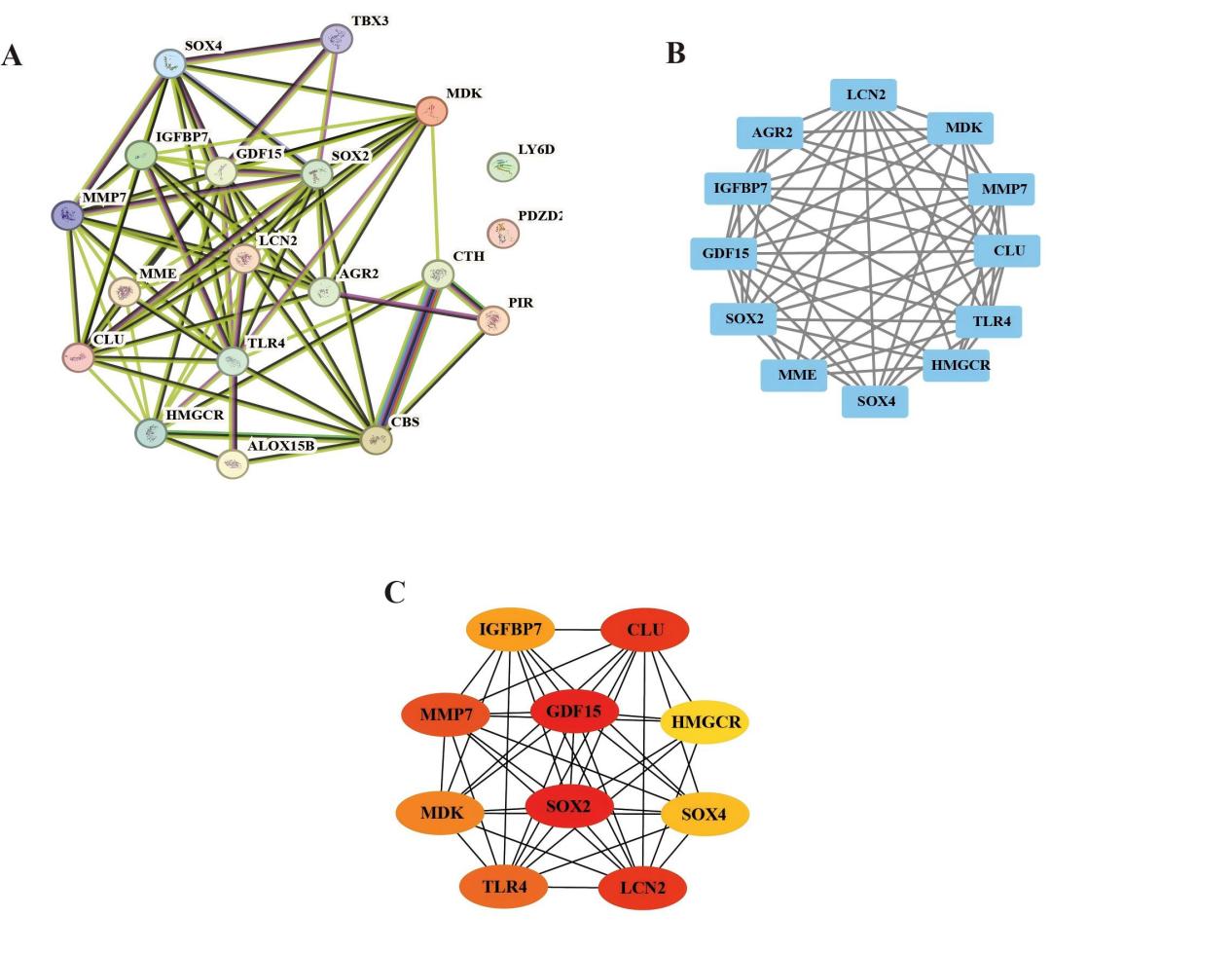


**Supplementary Figure 2 PPI network and hub genes of the aging-related differential genes.** **(A)** The PPI network of the aging-related differentially expressed genes. **(B)** Subnetwork of protein interactions among the aging-related differentially expressed genes. **(C)** The ten hub genes among the aging-related differentially expressed genes. PPI, Protein-protein interaction.
