# Supplementary figures and images for: The role of age-related genes in idiopathic pulmonary fibrosis and molecular docking analysis of their drug targets
Source: Front Immunol. 2026 Jan 5;16:1697013. doi: 10.3389/fimmu.2025.1697013 (PMC12812732; doi:10.3389/fimmu.2025.1697013)

CLU


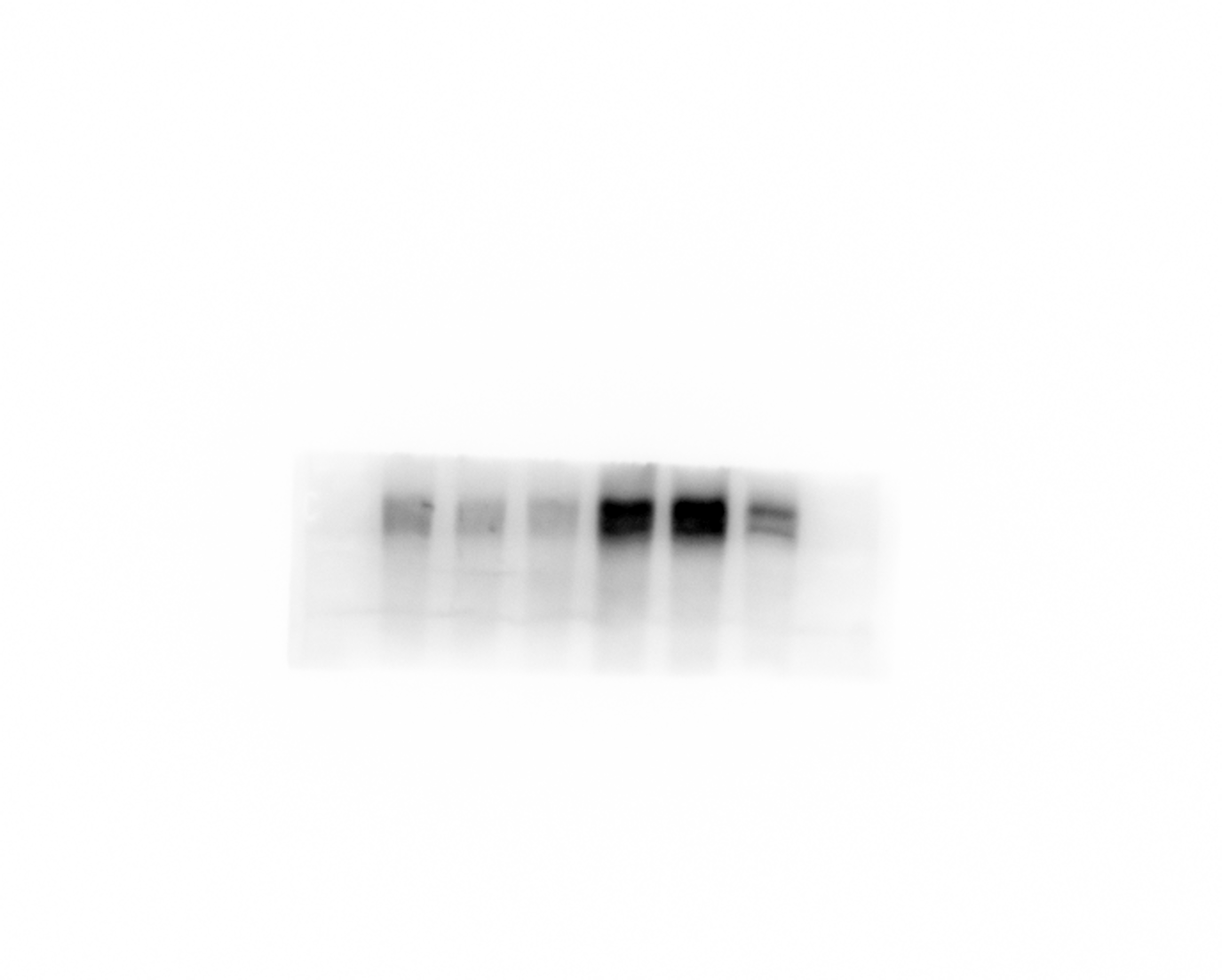


LCN2


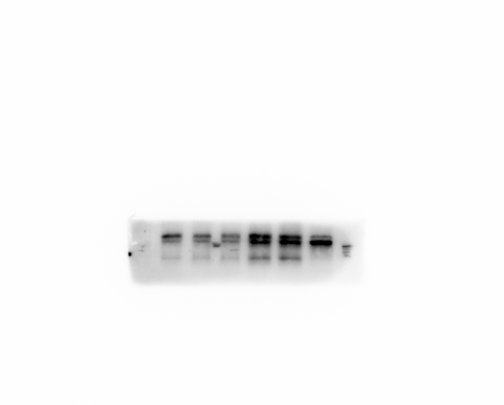


β-actin


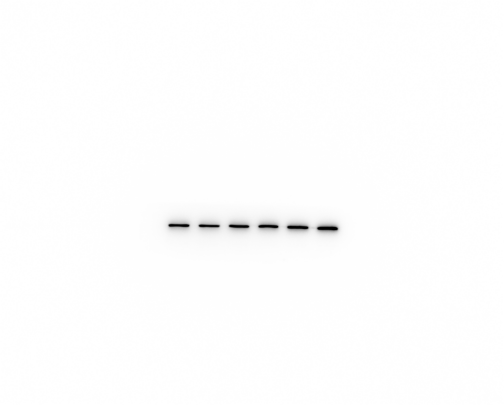


GAPDH


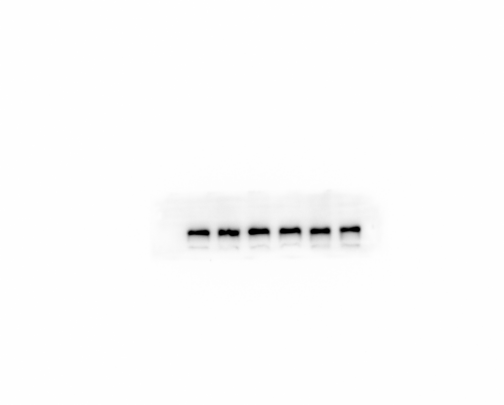

Supplement: Supplementary file 2 [file DataSheet2.docx]
