# Supplementary material for: The role of age-related genes in idiopathic pulmonary fibrosis and molecular docking analysis of their drug targets
Source: Front Immunol. 2026 Jan 5;16:1697013. doi: 10.3389/fimmu.2025.1697013 (PMC12812732; doi:10.3389/fimmu.2025.1697013)
Supplement: Supplementary file 3 [file Table1.docx]

**Supplementary table1:** Differentially expressed genes in IPF and **normal samples**.

| id | logFC | AveExpr | t | P.Value | adj.P.Val | B |
| --- | --- | --- | --- | --- | --- | --- |
| MMP1 | 2.21655164 | 1.86430315 | 4.768999867 | 6.90E-06 | 0.000133428 | 3.4513397 |
| MMP7 | 2.042584315 | 5.683870877 | 6.660204274 | 1.95E-09 | 1.77E-07 | 11.29287374 |
| SFRP2 | 1.77242473 | 3.208028927 | 6.771562491 | 1.17E-09 | 1.14E-07 | 11.78750882 |
| CXCL14 | 1.698490357 | 3.858920227 | 6.652470118 | 2.02E-09 | 1.82E-07 | 11.25862031 |
| COMP | 1.647659194 | 3.905589019 | 8.22882257 | 1.19E-12 | 4.68E-10 | 18.44743556 |
| IL13RA2 | 1.630296192 | 3.291245027 | 7.275972672 | 1.11E-10 | 1.47E-08 | 14.05836266 |
| S100A2 | 1.605565593 | 4.163433909 | 4.425756487 | 2.63E-05 | 0.000385936 | 2.181453526 |
| CCL19 | 1.574258037 | 4.622190756 | 7.357907931 | 7.56E-11 | 1.06E-08 | 14.43134241 |
| CXCL13 | 1.489028586 | 2.852568026 | 5.451297017 | 4.15E-07 | 1.36E-05 | 6.135212399 |
| LCN2 | 1.478934658 | 4.474537007 | 5.231680146 | 1.05E-06 | 2.91E-05 | 5.250144738 |
| CTHRC1 | 1.456621261 | 2.537722938 | 6.841155846 | 8.46E-10 | 8.39E-08 | 12.09796002 |
| KRT5 | 1.433663851 | 3.398272737 | 4.670345586 | 1.02E-05 | 0.000180722 | 3.080384208 |
| ASPN | 1.410984622 | 4.858934935 | 6.093730976 | 2.53E-08 | 1.39E-06 | 8.822691721 |
| MSMB | 1.399431501 | 2.282976291 | 2.8192595 | 0.005891958 | 0.027433837 | -2.840525149 |
| COL10A1 | 1.393974003 | 3.317615909 | 5.998828465 | 3.86E-08 | 1.98E-06 | 8.417340714 |
| THY1 | 1.363120329 | 2.627052469 | 9.292814814 | 6.92E-15 | 1.53E-11 | 23.42484547 |
| LGALS7 | 1.350258897 | 2.609058487 | 4.695754819 | 9.23E-06 | 0.000167624 | 3.175479174 |
| SPP1 | 1.348050762 | 1.262921997 | 4.008562425 | 0.000123866 | 0.001320543 | 0.721726993 |
| IGJ | 1.305995574 | 4.251167066 | 4.348681345 | 3.53E-05 | 0.000488485 | 1.904574708 |
| LGALS2 | 1.300828375 | 1.827080173 | 5.41676813 | 4.81E-07 | 1.53E-05 | 5.994824784 |
| HS6ST2 | 1.298540118 | 1.172460512 | 7.67859734 | 1.65E-11 | 2.98E-09 | 15.90026433 |
| FHL2 | 1.290621038 | 2.607304056 | 7.990128672 | 3.74E-12 | 1.03E-09 | 17.33895859 |
| GPR87 | 1.218661624 | 1.876048277 | 4.576866424 | 1.47E-05 | 0.000244193 | 2.733261841 |
| CHIT1 | 1.171565718 | 3.149856765 | 3.753008387 | 0.000305367 | 0.002765334 | -0.122607019 |
| SERPIND1 | 1.15360271 | 1.256293717 | 6.100030597 | 2.46E-08 | 1.37E-06 | 8.849692388 |
| TMPRSS4 | 1.137895147 | 2.05353245 | 4.641478251 | 1.14E-05 | 0.000197828 | 2.972729066 |
| SULF1 | 1.131825495 | 3.451726032 | 6.149543984 | 1.97E-08 | 1.15E-06 | 9.062307143 |
| COL14A1 | 1.110387659 | 2.910260249 | 6.970603266 | 4.64E-10 | 5.15E-08 | 12.67798874 |
| COL1A1 | 1.109563179 | 3.631676324 | 6.119972361 | 2.25E-08 | 1.28E-06 | 8.935239744 |
| FNDC1 | 1.082336336 | 2.643829779 | 6.60451244 | 2.52E-09 | 2.16E-07 | 11.04652239 |
| TDO2 | 1.08071664 | 2.70417223 | 6.440918012 | 5.31E-09 | 4.15E-07 | 10.32703574 |
| CP | 1.070807282 | 1.573630154 | 3.794235346 | 0.000264661 | 0.002474361 | 0.010900772 |
| CDH3 | 1.06062071 | 2.999874489 | 6.556518857 | 3.13E-09 | 2.63E-07 | 10.83479129 |
| COL15A1 | 1.044359417 | 3.164372817 | 5.79227102 | 9.56E-08 | 4.22E-06 | 7.544624853 |
| KRT14 | 1.04223935 | 1.720551868 | 5.713505615 | 1.35E-07 | 5.55E-06 | 7.215464621 |
| MXRA5 | 1.040830438 | 2.843310769 | 7.834132876 | 7.88E-12 | 1.71E-09 | 16.61727662 |
| BDKRB2 | 1.030334667 | 3.113973388 | 4.848030421 | 5.04E-06 | 0.00010546 | 3.751832032 |
| CCDC78 | 1.029849602 | 1.883920126 | 3.885662601 | 0.000192043 | 0.001883588 | 0.31072398 |
| CLIC6 | 1.028579872 | 4.164930594 | 5.763330248 | 1.08E-07 | 4.56E-06 | 7.423441317 |
| C15orf48 | 1.02791994 | 3.621405408 | 5.2107697 | 1.14E-06 | 3.11E-05 | 5.166870216 |
| MDK | 1.023878318 | 1.313111962 | 9.731471791 | 8.24E-16 | 5.35E-12 | 25.48141273 |
| IGHA1 | 1.01484185 | 3.35851334 | 4.518703679 | 1.84E-05 | 0.000293927 | 2.519485323 |
| SFRP4 | 1.01345967 | 3.971208563 | 4.806202456 | 5.95E-06 | 0.000119483 | 3.592429255 |
| COL3A1 | 1.003172869 | 6.381181878 | 5.328052378 | 7.00E-07 | 2.09E-05 | 5.63621132 |
| ALDH1A3 | 0.990154465 | 2.946164545 | 6.036808879 | 3.26E-08 | 1.74E-06 | 8.579244088 |
| KRT15 | 0.982868784 | 3.204752193 | 3.204096704 | 0.001861357 | 0.011322339 | -1.794451057 |
| SCGB3A1 | 0.978083742 | 6.781401588 | 3.707264059 | 0.000357487 | 0.003101088 | -0.269495876 |
| SCG5 | 0.974513693 | 0.796368262 | 6.423581687 | 5.74E-09 | 4.44E-07 | 10.25116962 |
| CXCL12 | 0.973665678 | 4.118073425 | 6.824901442 | 9.13E-10 | 8.98E-08 | 12.02536138 |
| C9orf24 | 0.962893499 | 4.83433239 | 2.683520003 | 0.008636389 | 0.036413036 | -3.182870529 |
| MMP10 | 0.961427242 | 2.280117941 | 3.890927345 | 0.000188502 | 0.001854773 | 0.328144546 |
| LRRN1 | 0.951222522 | 1.416162659 | 6.39303416 | 6.59E-09 | 4.87E-07 | 10.11767221 |
| SCGB1A1 | 0.921765672 | 5.84711401 | 3.896772329 | 0.000184643 | 0.00182618 | 0.347504742 |
| LTB | 0.921108522 | 3.960663804 | 6.322729221 | 9.06E-09 | 6.35E-07 | 9.811331189 |
| VCAM1 | 0.92057955 | 3.134619589 | 4.590350925 | 1.40E-05 | 0.000235008 | 2.783067229 |
| FAM81B | 0.916176485 | 3.370088125 | 3.15871142 | 0.002143215 | 0.012633588 | -1.923448146 |
| CXCL6 | 0.910827764 | 2.34092223 | 3.31708102 | 0.001302815 | 0.008609848 | -1.466998467 |
| DIO2 | 0.910578535 | 2.262904142 | 5.118633947 | 1.67E-06 | 4.26E-05 | 4.802089226 |
| CTSG | 0.906894747 | 2.624118123 | 4.847566966 | 5.04E-06 | 0.000105486 | 3.750061406 |
| CHRDL2 | 0.902213059 | 2.967774388 | 4.497193469 | 2.00E-05 | 0.000314718 | 2.440860309 |
| UGT1A6 | 0.90011034 | 1.824877077 | 4.706008078 | 8.86E-06 | 0.000163027 | 3.213940903 |
| TSPAN1 | 0.897987813 | 3.774830124 | 4.288747724 | 4.42E-05 | 0.000581151 | 1.691470394 |
| KIAA0125 | 0.896734312 | 2.967293466 | 4.425131137 | 2.64E-05 | 0.000385936 | 2.179194444 |
| GEM | 0.895236352 | 3.53278853 | 6.128202768 | 2.17E-08 | 1.24E-06 | 8.970580282 |
| SOX2 | 0.886019912 | 0.787282005 | 3.335893792 | 0.001226704 | 0.008221796 | -1.411610022 |
| DYNLRB2 | 0.87601266 | 3.638500405 | 2.623460478 | 0.010186499 | 0.041351009 | -3.329754175 |
| IL4I1 | 0.864763986 | 2.359549934 | 4.781568914 | 6.57E-06 | 0.000128833 | 3.498934603 |
| ADRA2A | 0.861268131 | 2.788562928 | 8.156640614 | 1.68E-12 | 6.30E-10 | 18.11174373 |
| ST6GALNAC1 | 0.855634245 | 2.851044525 | 3.904502892 | 0.000179655 | 0.001785007 | 0.373142241 |
| LY6D | 0.853267579 | 2.137244187 | 3.727954254 | 0.000332944 | 0.002947245 | -0.203221389 |
| HDC | 0.841045433 | 4.161191796 | 4.708745682 | 8.77E-06 | 0.000161843 | 3.224218699 |
| CD24 | 0.84041637 | 2.321520274 | 4.495316056 | 2.02E-05 | 0.000316237 | 2.434009158 |
| COLEC11 | 0.839163602 | 2.562483107 | 5.607648086 | 2.13E-07 | 8.03E-06 | 6.776409641 |
| TNFRSF17 | 0.827120666 | 1.800125908 | 3.275001333 | 0.001489379 | 0.009557867 | -1.589999784 |
| CCR7 | 0.826149946 | 3.320331167 | 3.833207974 | 0.000230976 | 0.002203858 | 0.138078497 |
| CAPSL | 0.824177558 | 3.523162673 | 2.587905132 | 0.01121876 | 0.044593453 | -3.415364346 |
| THBS4 | 0.819330293 | 2.395151937 | 6.371949084 | 7.26E-09 | 5.29E-07 | 10.02566467 |
| GZMK | 0.816676003 | 3.384734381 | 5.488617665 | 3.54E-07 | 1.21E-05 | 6.287452677 |
| CTSK | 0.810658302 | 4.580536418 | 4.449846796 | 2.40E-05 | 0.000360888 | 2.268635699 |
| PPAP2C | 0.802872014 | 1.85072055 | 4.185222999 | 6.50E-05 | 0.000789689 | 1.328000554 |
| CXCL9 | 0.796023765 | 3.867729124 | 2.958599822 | 0.003926874 | 0.020092349 | -2.474432289 |
| CCL11 | 0.793465335 | 2.523716776 | 4.436993137 | 2.52E-05 | 0.000374839 | 2.222080933 |
| CCR5 | 0.790131601 | 4.185189161 | 5.77114287 | 1.05E-07 | 4.48E-06 | 7.456127778 |
| AGR2 | 0.777199239 | 2.804165261 | 3.831541435 | 0.000232328 | 0.002213512 | 0.132620976 |
| CPXM2 | 0.766342383 | 3.594094906 | 4.86612076 | 4.68E-06 | 9.95E-05 | 3.821023182 |
| VTCN1 | 0.76246896 | 1.517578276 | 2.962543462 | 0.003881292 | 0.019916167 | -2.463858559 |
| ASB2 | 0.762034309 | 2.358207056 | 5.646106625 | 1.80E-07 | 7.07E-06 | 6.935471191 |
| TGFB3 | 0.761352199 | 3.032915601 | 6.903307238 | 6.35E-10 | 6.87E-08 | 12.37604065 |
| CRIP1 | 0.753646505 | 4.146897744 | 6.355249996 | 7.82E-09 | 5.65E-07 | 9.952876511 |
| MS4A2 | 0.753254898 | 3.185539957 | 4.085338931 | 9.38E-05 | 0.001055424 | 0.983008302 |
| DMBT1 | 0.751895986 | 6.060926536 | 5.111756665 | 1.72E-06 | 4.36E-05 | 4.77500301 |
| NELL2 | 0.739267628 | 2.386821468 | 3.680993055 | 0.000391134 | 0.003324124 | -0.353255425 |
| LRRC46 | 0.738522864 | 2.819834338 | 2.935862551 | 0.004199452 | 0.021104521 | -2.535169265 |
| CCDC80 | 0.733741531 | 2.857823557 | 4.266370491 | 4.81E-05 | 0.000620629 | 1.612404459 |
| CAPS | 0.73087142 | 3.801078932 | 2.583254388 | 0.011360547 | 0.044978067 | -3.426487994 |
| CCL21 | 0.728718963 | 3.522799876 | 5.434013011 | 4.47E-07 | 1.44E-05 | 6.064882889 |
| MFAP2 | 0.725276395 | 1.900529703 | 5.899042673 | 5.99E-08 | 2.84E-06 | 7.994063835 |
| SERPINF1 | 0.724902817 | 3.704071083 | 5.785919345 | 9.83E-08 | 4.28E-06 | 7.518004846 |
| TMEM45A | 0.721846318 | 2.741315998 | 3.391575534 | 0.001025167 | 0.007145906 | -1.246244413 |
| TNS4 | 0.721509272 | 2.032316895 | 3.308214328 | 0.001340202 | 0.00879872 | -1.493018737 |
| CCL5 | 0.721245162 | 3.717358685 | 4.433298861 | 2.56E-05 | 0.000378774 | 2.208716562 |
| KCNN4 | 0.717847023 | 1.763753138 | 6.032636663 | 3.32E-08 | 1.76E-06 | 8.561437658 |
| PLA2G7 | 0.715858877 | 3.76757757 | 3.212540385 | 0.001812897 | 0.011125354 | -1.770290128 |
| SLITRK6 | 0.715482008 | 3.184334087 | 3.142706495 | 0.002251757 | 0.013159909 | -1.968587098 |
| TNNI2 | 0.710929523 | 1.86354834 | 6.010822314 | 3.66E-08 | 1.91E-06 | 8.468421567 |
| STEAP1 | 0.709772156 | 2.650977025 | 4.280379128 | 4.56E-05 | 0.000594252 | 1.661869426 |
| TNC | 0.709135494 | 1.589069396 | 5.22313972 | 1.09E-06 | 2.99E-05 | 5.216111549 |
| PSD3 | 0.704875397 | 2.201252032 | 8.026126813 | 3.15E-12 | 9.45E-10 | 17.50582458 |
| FCRL5 | 0.703088889 | 2.48242432 | 3.707857742 | 0.00035676 | 0.003096845 | -0.267597976 |
| LRRC17 | 0.702145283 | 1.283995781 | 5.661616497 | 1.69E-07 | 6.71E-06 | 6.999764854 |
| C1orf54 | 0.700285006 | 4.353228938 | 7.364932094 | 7.31E-11 | 1.03E-08 | 14.46336448 |
| GJB2 | 0.696373215 | 1.866425954 | 4.429058216 | 2.60E-05 | 0.000382778 | 2.193384455 |
| SCRG1 | 0.696027672 | 2.461134176 | 3.805847626 | 0.000254163 | 0.002391714 | 0.048696434 |
| CHST6 | 0.69576615 | 1.923423551 | 3.067255571 | 0.002835865 | 0.015785059 | -2.178892141 |
| BCHE | 0.695750986 | 2.465604667 | 4.374974301 | 3.19E-05 | 0.000450454 | 1.998673242 |
| CLU | 0.693047104 | 2.717056072 | 5.820851187 | 8.44E-08 | 3.82E-06 | 7.66456838 |
| SLN | 0.685972112 | 3.036534049 | 2.905444597 | 0.004591409 | 0.022567765 | -2.615817533 |
| FANK1 | 0.684873223 | 2.684595859 | 3.426044702 | 0.000916478 | 0.006557234 | -1.142815509 |
| CD3D | 0.682333305 | 0.929787252 | 5.318702733 | 7.28E-07 | 2.14E-05 | 5.598595341 |
| SPAG6 | 0.677860802 | 2.617886125 | 2.87213384 | 0.005059076 | 0.024278332 | -2.703335924 |
| SOX4 | 0.677517151 | 2.146105254 | 7.798166815 | 9.36E-12 | 1.96E-09 | 16.45123718 |
| MMP11 | 0.677130182 | 1.875332328 | 3.460504963 | 0.000818732 | 0.005983146 | -1.038609607 |
| APOE | 0.676718909 | 3.137687252 | 4.737752497 | 7.82E-06 | 0.000146934 | 3.333340214 |
| IGFBP7 | 0.673751626 | 4.137596215 | 8.00732186 | 3.44E-12 | 9.72E-10 | 17.41864143 |
| GSTA1 | 0.672204834 | 3.040294331 | 2.962884303 | 0.003877376 | 0.019916167 | -2.462944145 |
| CLDN1 | 0.67091358 | 2.590088414 | 4.376967163 | 3.17E-05 | 0.00044794 | 2.005820424 |
| IGFBP2 | 0.669255433 | 2.305978434 | 4.735512952 | 7.89E-06 | 0.000147999 | 3.324900855 |
| WNT10A | 0.666182485 | 2.071059065 | 6.242853785 | 1.30E-08 | 8.31E-07 | 9.46485857 |
| CPZ | 0.663827907 | 1.239055025 | 4.923054077 | 3.72E-06 | 8.25E-05 | 4.039753845 |
| HBA1 | 0.66354643 | 3.854262079 | 3.118049267 | 0.002429036 | 0.01394494 | -2.037767671 |
| CFH | 0.652559869 | 4.821924322 | 6.240488747 | 1.31E-08 | 8.36E-07 | 9.454625891 |
| ZNF521 | 0.652478319 | 2.361196885 | 4.862755548 | 4.75E-06 | 0.000100703 | 3.808140684 |
| COL1A2 | 0.650145862 | 4.087831588 | 4.457209868 | 2.33E-05 | 0.000354957 | 2.295342829 |
| PLN | 0.64793018 | 4.859449335 | 3.749491785 | 0.000309102 | 0.002793316 | -0.133945864 |
| KLHL13 | 0.647141362 | 1.234757673 | 5.791793495 | 9.58E-08 | 4.22E-06 | 7.542623074 |
| PTGDS | 0.646581114 | 5.320495076 | 6.640245713 | 2.14E-09 | 1.90E-07 | 11.20450742 |
| FCER1A | 0.642911829 | 3.060830172 | 3.823590473 | 0.000238887 | 0.002267676 | 0.106606982 |
| CNN1 | 0.642183509 | 4.408161795 | 4.15446758 | 7.28E-05 | 0.000859688 | 1.221170318 |
| IGFL2 | 0.638008145 | 2.000412881 | 3.808229052 | 0.00025206 | 0.00237709 | 0.056457823 |
| HMCN1 | 0.637094233 | 3.438054302 | 6.631975714 | 2.22E-09 | 1.96E-07 | 11.16791819 |
| GDF15 | 0.636243404 | 1.246956582 | 4.50076525 | 1.97E-05 | 0.000311548 | 2.453899627 |
| VMO1 | 0.636031866 | 3.144026067 | 4.968196779 | 3.10E-06 | 7.10E-05 | 4.214224259 |
| TNFRSF21 | 0.635860062 | 2.375423283 | 6.882854325 | 6.98E-10 | 7.23E-08 | 12.2844448 |
| CYP24A1 | 0.63559098 | 0.82784425 | 3.525800431 | 0.000659879 | 0.005055567 | -0.838977779 |
| CYP2F1 | 0.635544071 | 1.633856131 | 3.075513016 | 0.002765686 | 0.015468814 | -2.156077465 |
| CD19 | 0.633516354 | 2.095819746 | 3.457709227 | 0.00082628 | 0.006028131 | -1.047093563 |
| LXN | 0.633251758 | 3.676845898 | 4.897505173 | 4.13E-06 | 8.96E-05 | 3.941416185 |
| CCDC17 | 0.630291936 | 3.09846793 | 2.561589427 | 0.012042584 | 0.047174959 | -3.478078569 |
| RGS5 | 0.62790729 | 3.834728142 | 5.133531986 | 1.57E-06 | 4.08E-05 | 4.860833544 |
| COL17A1 | 0.627887852 | 2.239251332 | 4.070214408 | 9.91E-05 | 0.001106518 | 0.931266615 |
| CRISPLD1 | 0.625184356 | 1.741154045 | 6.304439813 | 9.84E-09 | 6.74E-07 | 9.731848483 |
| COL6A3 | 0.624500555 | 4.479232352 | 4.398658921 | 2.92E-05 | 0.000417962 | 2.083751811 |
| PSCA | 0.622980039 | 1.579196183 | 2.631233935 | 0.009972594 | 0.04071176 | -3.310903569 |
| ITGBL1 | 0.622399665 | 2.751410823 | 3.74312143 | 0.000315979 | 0.002835732 | -0.154466517 |
| MMP12 | 0.62237392 | 1.759408857 | 2.900812717 | 0.004653966 | 0.022772167 | -2.628037166 |
| PTPRZ1 | 0.62219699 | 0.959957945 | 2.714807903 | 0.007916757 | 0.034109825 | -3.105229083 |
| CPA3 | 0.620930132 | 5.041488394 | 3.270747582 | 0.001509571 | 0.009647288 | -1.602365007 |
| RHOV | 0.616018009 | 1.227244201 | 2.755505994 | 0.007062417 | 0.03136544 | -3.003094141 |
| RAMP1 | 0.610138941 | 3.254052479 | 5.083601092 | 1.93E-06 | 4.78E-05 | 4.664320959 |
| SYT8 | 0.60942747 | 2.235762805 | 3.588304233 | 0.000535473 | 0.004266041 | -0.645237888 |
| ICOS | 0.607082635 | 2.229746378 | 4.828025009 | 5.46E-06 | 0.000111566 | 3.675491854 |
| FRZB | 0.605759352 | 3.132744061 | 5.301215916 | 7.83E-07 | 2.26E-05 | 5.52833377 |
| TSHZ2 | 0.602349804 | 2.409363336 | 7.348428677 | 7.91E-11 | 1.09E-08 | 14.38813959 |
| UCP2 | 0.600625984 | 2.518410039 | 6.740902853 | 1.34E-09 | 1.27E-07 | 11.65105803 |
| THBS2 | 0.599958339 | 1.968646027 | 3.20834016 | 0.001836854 | 0.011208166 | -1.782315062 |
| AEBP1 | 0.599067396 | 3.136894944 | 5.622578297 | 2.00E-07 | 7.58E-06 | 6.838098248 |
| KLHDC7B | 0.598247076 | 1.767522897 | 6.606596076 | 2.49E-09 | 2.16E-07 | 11.05572667 |
| PCP4 | 0.597673497 | 0.848998118 | 3.184033155 | 0.0019814 | 0.011862821 | -1.851658158 |
| GBP5 | 0.596228087 | 3.885788645 | 3.453892114 | 0.000836692 | 0.006086992 | -1.058668515 |
| HLA-DQB2 | 0.595143728 | 4.262729025 | 4.000603943 | 0.000127466 | 0.001351246 | 0.694840196 |
| LTBP2 | 0.594140493 | 3.711929809 | 5.468949053 | 3.85E-07 | 1.31E-05 | 6.207154909 |
| PDLIM4 | 0.590121678 | 1.833931121 | 4.532645782 | 1.75E-05 | 0.000282318 | 2.570573237 |
| SPINK5 | 0.590098157 | 3.028308003 | 3.189054152 | 0.001950699 | 0.011722247 | -1.837368685 |
| CD2 | 0.588784195 | 4.432271776 | 4.062906696 | 0.000101764 | 0.001121822 | 0.906313975 |
| GREM1 | 0.588118543 | 2.028186104 | 2.637602294 | 0.009800393 | 0.040192058 | -3.29542468 |
| EYA2 | 0.586466512 | 2.342083014 | 2.803636871 | 0.006161073 | 0.02837867 | -2.880651433 |
| PDZD2 | -0.585313846 | 3.001076002 | -5.464172341 | 3.93E-07 | 1.32E-05 | 6.187675503 |
| CLEC4D | -0.585415621 | 1.949400886 | -3.34628599 | 0.001186469 | 0.008022827 | -1.380908567 |
| WISP2 | -0.586800595 | 3.104884606 | -3.343747779 | 0.00119618 | 0.008069402 | -1.388414023 |
| SAP30 | -0.587551041 | 0.821209167 | -6.861383479 | 7.71E-10 | 7.70E-08 | 12.18837911 |
| CA3 | -0.588580131 | 3.903782569 | -2.623917236 | 0.010173816 | 0.041312439 | -3.328647861 |
| SLC19A2 | -0.588646022 | 2.547151687 | -3.077576749 | 0.002748401 | 0.015383935 | -2.150367768 |
| TSC22D3 | -0.589043287 | 2.752590661 | -5.313903648 | 7.42E-07 | 2.17E-05 | 5.57930075 |
| NFKBIZ | -0.591405107 | 3.53696035 | -4.467786727 | 2.24E-05 | 0.000344067 | 2.33375618 |
| REPS2 | -0.5914619 | 1.835319388 | -8.98913826 | 3.02E-14 | 2.61E-11 | 22.00088235 |
| CD274 | -0.591752756 | 2.700415676 | -5.047276021 | 2.24E-06 | 5.42E-05 | 4.522023835 |
| CMTM2 | -0.592953457 | 2.145565108 | -4.409906766 | 2.80E-05 | 0.000404905 | 2.124259597 |
| TBX3 | -0.593290796 | 1.35908787 | -6.283291338 | 1.08E-08 | 7.25E-07 | 9.640050727 |
| KLF15 | -0.598751891 | 2.003164611 | -8.2625068 | 1.01E-12 | 4.10E-10 | 18.60421933 |
| ADORA3 | -0.599066243 | 1.989258203 | -5.412375719 | 4.90E-07 | 1.54E-05 | 5.976998413 |
| TTN | -0.599307958 | 1.402963534 | -7.706443013 | 1.45E-11 | 2.77E-09 | 16.02843061 |
| CDKL2 | -0.60332582 | 2.821961723 | -6.487853995 | 4.29E-09 | 3.44E-07 | 10.53280522 |
| ALPP | -0.604901414 | 1.633517111 | -7.577601369 | 2.67E-11 | 4.29E-09 | 15.43619228 |
| SLCO2A1 | -0.606262488 | 4.62949874 | -3.619720206 | 0.000481671 | 0.003921616 | -0.546894916 |
| WWC2 | -0.60673139 | 1.813688531 | -6.128582983 | 2.17E-08 | 1.24E-06 | 8.972213351 |
| OLFM4 | -0.609109757 | 1.086006145 | -7.312045776 | 9.38E-11 | 1.27E-08 | 14.22244568 |
| PTPRG | -0.611370921 | 1.967739545 | -6.898213126 | 6.50E-10 | 6.97E-08 | 12.35321963 |
| SLC39A8 | -0.614312071 | 2.589304191 | -3.64224415 | 0.000446295 | 0.003682078 | -0.475993555 |
| SRPX | -0.614467306 | 2.681175847 | -5.564017426 | 2.57E-07 | 9.42E-06 | 6.596589317 |
| SIGLEC5 | -0.615892394 | 2.479372559 | -4.745835347 | 7.57E-06 | 0.000142889 | 3.363818994 |
| MMP25 | -0.617036232 | 2.331994815 | -4.745116139 | 7.59E-06 | 0.000143089 | 3.361105744 |
| TEK | -0.624329781 | 3.410565051 | -5.456514127 | 4.06E-07 | 1.35E-05 | 6.156463103 |
| PLLP | -0.627571209 | 2.176558229 | -4.937242437 | 3.51E-06 | 7.87E-05 | 4.09449178 |
| ADRB2 | -0.633445955 | 3.444276936 | -7.246179466 | 1.28E-10 | 1.64E-08 | 13.92299802 |
| PHACTR3 | -0.63359487 | 2.836687272 | -3.850371858 | 0.000217475 | 0.002099722 | 0.194385386 |
| HMGCR | -0.638347371 | 1.789980372 | -7.83221197 | 7.95E-12 | 1.71E-09 | 16.60840518 |
| SEC14L4 | -0.638567973 | 2.236320095 | -4.235578356 | 5.39E-05 | 0.000679291 | 1.504055274 |
| CRTAC1 | -0.639727086 | 2.694647926 | -4.708571063 | 8.77E-06 | 0.000161843 | 3.223563017 |
| BTNL3 | -0.643600707 | 2.001455754 | -4.420547793 | 2.69E-05 | 0.000391424 | 2.162643329 |
| NEBL | -0.647984708 | 2.137205625 | -9.423401804 | 3.67E-15 | 1.19E-11 | 24.03728537 |
| TNNC1 | -0.654089363 | 3.211997023 | -4.1372123 | 7.76E-05 | 0.000902769 | 1.161467352 |
| ADHFE1 | -0.655255636 | 2.843021461 | -7.248711457 | 1.26E-10 | 1.64E-08 | 13.93449658 |
| NPR1 | -0.65553825 | 2.355853577 | -6.719821574 | 1.48E-09 | 1.38E-07 | 11.55735166 |
| APOLD1 | -0.658272161 | 3.949810494 | -3.486646657 | 0.00075123 | 0.005600158 | -0.959026322 |
| ODAM | -0.660081478 | 1.209384262 | -5.421894166 | 4.71E-07 | 1.50E-05 | 6.01563779 |
| ADM | -0.666394344 | 1.876631059 | -3.52520786 | 0.00066118 | 0.005062546 | -0.840802229 |
| FAM46B | -0.670090992 | 1.917125813 | -9.244102882 | 8.76E-15 | 1.53E-11 | 23.19638613 |
| CBS | -0.672131879 | 1.174610288 | -8.863069076 | 5.56E-14 | 3.80E-11 | 21.41014213 |
| TIMP3 | -0.674009598 | 3.786677101 | -4.836998446 | 5.26E-06 | 0.000108309 | 3.709711389 |
| APOH | -0.674994935 | -1.414342476 | -4.780989516 | 6.58E-06 | 0.000128936 | 3.496738974 |
| PKHD1L1 | -0.679364624 | 1.905520262 | -5.415841112 | 4.83E-07 | 1.53E-05 | 5.99106192 |
| MME | -0.683582016 | 1.977906119 | -5.037209916 | 2.34E-06 | 5.60E-05 | 4.482692138 |
| FUT1 | -0.683795321 | 2.550105994 | -5.506575057 | 3.28E-07 | 1.15E-05 | 6.360889135 |
| LEPREL1 | -0.685669882 | 1.720480069 | -5.044259474 | 2.27E-06 | 5.46E-05 | 4.510232563 |
| NUDT16 | -0.686938342 | 2.299486666 | -9.01970797 | 2.60E-14 | 2.59E-11 | 22.14417679 |
| LGI3 | -0.688221888 | 2.504554795 | -5.691446424 | 1.48E-07 | 6.00E-06 | 7.123653395 |
| VEPH1 | -0.690602859 | 2.542502009 | -4.504936051 | 1.94E-05 | 0.000307325 | 2.469134032 |
| TLR4 | -0.691020219 | 2.533189523 | -7.974257204 | 4.03E-12 | 1.05E-09 | 17.26542537 |
| RPS6KA2 | -0.691886053 | 3.574287321 | -8.282296275 | 9.19E-13 | 3.85E-10 | 18.69636681 |
| VNN2 | -0.69284531 | 3.740182235 | -3.526042673 | 0.000659348 | 0.005054481 | -0.838231879 |
| ABCC8 | -0.695194063 | 1.915467058 | -4.425630444 | 2.63E-05 | 0.000385936 | 2.180998181 |
| MCCC1 | -0.696404383 | 2.558893019 | -9.040537886 | 2.35E-14 | 2.54E-11 | 22.24182538 |
| SLC1A1 | -0.70355443 | 2.5453523 | -6.347314447 | 8.11E-09 | 5.82E-07 | 9.918312087 |
| FZD5 | -0.703924779 | 1.750021128 | -7.975411837 | 4.01E-12 | 1.05E-09 | 17.27077405 |
| AASS | -0.705569287 | 2.006083625 | -7.963321957 | 4.25E-12 | 1.08E-09 | 17.21477548 |
| CACNA2D2 | -0.70570873 | 3.802226463 | -4.141813289 | 7.63E-05 | 0.000891754 | 1.177370185 |
| SIGLEC10 | -0.707106561 | 2.157177618 | -6.751460778 | 1.28E-09 | 1.23E-07 | 11.69802367 |
| PLA1A | -0.710557647 | 2.623732126 | -4.797211281 | 6.17E-06 | 0.000122712 | 3.558271003 |
| NFE2 | -0.711548547 | 0.401377266 | -5.423209786 | 4.68E-07 | 1.50E-05 | 6.020981142 |
| PGC | -0.714083933 | 4.199563981 | -2.757400231 | 0.007024782 | 0.031305361 | -2.9983091 |
| NOSTRIN | -0.725942977 | 3.211750845 | -5.93874354 | 5.03E-08 | 2.45E-06 | 8.16210129 |
| TMTC1 | -0.728361785 | 2.163882506 | -7.758453688 | 1.13E-11 | 2.23E-09 | 16.26806158 |
| TACC2 | -0.730262148 | 2.872288272 | -6.582926735 | 2.78E-09 | 2.37E-07 | 10.9512279 |
| ANXA3 | -0.733061925 | 3.259708167 | -4.322227564 | 3.90E-05 | 0.000530707 | 1.810274484 |
| CDH19 | -0.736183809 | 1.451580505 | -6.43605086 | 5.43E-09 | 4.22E-07 | 10.30572889 |
| ECHDC3 | -0.738296863 | 2.72975397 | -7.421318883 | 5.60E-11 | 8.09E-09 | 14.72068467 |
| GPM6A | -0.739397065 | 2.734707268 | -5.65320785 | 1.75E-07 | 6.90E-06 | 6.964897812 |
| PRX | -0.74001801 | 2.479111434 | -3.773050907 | 0.000284885 | 0.002620052 | -0.057834554 |
| SCN7A | -0.760995493 | 3.347587185 | -5.236951285 | 1.02E-06 | 2.86E-05 | 5.27116473 |
| TGFBR3 | -0.766962223 | 2.754475299 | -7.493272831 | 3.99E-11 | 6.09E-09 | 15.04970086 |
| PTPRB | -0.76864845 | 3.36029827 | -5.536773331 | 2.89E-07 | 1.04E-05 | 6.484650821 |
| SDPR | -0.776862942 | 3.699838022 | -6.040361503 | 3.21E-08 | 1.73E-06 | 8.594410258 |
| CLDN18 | -0.779366324 | 3.768111963 | -3.122362603 | 0.002397115 | 0.013811544 | -2.025697419 |
| GPM6B | -0.780134598 | 2.259949445 | -5.930212588 | 5.22E-08 | 2.52E-06 | 8.125951878 |
| USP53 | -0.797433649 | 2.441528643 | -8.642809964 | 1.61E-13 | 9.11E-11 | 20.37914605 |
| SCEL | -0.799379004 | 3.985974718 | -4.75960279 | 7.17E-06 | 0.000137892 | 3.41580478 |
| RNASE2 | -0.801515883 | 2.25312856 | -5.904035129 | 5.86E-08 | 2.79E-06 | 8.015167775 |
| DEFA4 | -0.802919158 | 1.688152083 | -6.297719717 | 1.01E-08 | 6.90E-07 | 9.702666184 |
| HIF3A | -0.810546389 | 1.836491008 | -9.284412478 | 7.21E-15 | 1.53E-11 | 23.3854382 |
| PIR | -0.81166719 | 1.331872314 | -9.223215512 | 9.70E-15 | 1.53E-11 | 23.09842613 |
| MPP3 | -0.816511546 | 1.952294481 | -7.975632697 | 4.01E-12 | 1.05E-09 | 17.27179717 |
| AOX1 | -0.820180332 | 2.658505933 | -6.881558764 | 7.02E-10 | 7.23E-08 | 12.27864557 |
| CHI3L2 | -0.823749153 | 2.19010153 | -3.934634073 | 0.000161417 | 0.00163252 | 0.47341253 |
| ZBTB16 | -0.828065193 | 4.087921085 | -2.947355898 | 0.004059559 | 0.020592748 | -2.504515927 |
| HSD17B6 | -0.843546699 | 3.699326727 | -5.164855104 | 1.38E-06 | 3.65E-05 | 4.984646397 |
| SERPINA3 | -0.847186651 | 2.054231884 | -4.606902364 | 1.31E-05 | 0.000221464 | 2.844324517 |
| TFCP2L1 | -0.847670606 | 2.881242527 | -7.829717303 | 8.05E-12 | 1.71E-09 | 16.59688448 |
| CD163 | -0.849411448 | 6.195617144 | -6.504480615 | 3.97E-09 | 3.21E-07 | 10.60582546 |
| STXBP6 | -0.854048449 | 1.799658919 | -5.046990816 | 2.25E-06 | 5.42E-05 | 4.520908836 |
| RNF182 | -0.854235912 | 0.561442306 | -6.976542149 | 4.52E-10 | 5.05E-08 | 12.70467704 |
| OLAH | -0.855668567 | 1.213890372 | -10.50046378 | 2.00E-17 | 2.59E-13 | 29.07356718 |
| GPX3 | -0.857330969 | 4.309741814 | -4.613946316 | 1.27E-05 | 0.000216366 | 2.870435686 |
| FCN3 | -0.869155837 | 4.899456357 | -2.624879413 | 0.010147147 | 0.041242832 | -3.326316832 |
| PEBP4 | -0.869555576 | 4.313199852 | -4.114768607 | 8.42E-05 | 0.000963527 | 1.084065805 |
| ACADL | -0.870477779 | 2.272997998 | -6.45181722 | 5.05E-09 | 3.98E-07 | 10.37477025 |
| MAOA | -0.882854904 | 3.755391801 | -7.336670611 | 8.36E-11 | 1.14E-08 | 14.33456949 |
| CYP3A5 | -0.886882256 | 2.242950342 | -5.107964772 | 1.75E-06 | 4.40E-05 | 4.760077159 |
| CTH | -0.896025337 | 0.702879697 | -8.462868391 | 3.85E-13 | 1.92E-10 | 19.53834006 |
| PDK4 | -0.901173633 | 2.990201703 | -5.693571142 | 1.47E-07 | 5.96E-06 | 7.13248934 |
| ALOX15B | -0.911825301 | 3.948187383 | -4.829536053 | 5.42E-06 | 0.000111068 | 3.681251496 |
| ST6GALNAC3 | -0.913135353 | 1.344951179 | -8.94331835 | 3.77E-14 | 2.88E-11 | 21.78613642 |
| TMEM100 | -0.919580047 | 4.091499695 | -2.941742085 | 0.004127342 | 0.020855124 | -2.519500596 |
| FAM105A | -0.93237036 | 2.467888643 | -7.69393634 | 1.54E-11 | 2.89E-09 | 15.97085445 |
| BTNL8 | -0.937569743 | 1.928445719 | -8.053166735 | 2.76E-12 | 8.75E-10 | 17.63124131 |
| KCNMB4 | -0.956327741 | 1.920641404 | -7.68040716 | 1.64E-11 | 2.98E-09 | 15.90859168 |
| FMO5 | -0.956955167 | 1.489640602 | -8.312952598 | 7.93E-13 | 3.43E-10 | 18.83916705 |
| CA2 | -0.980187818 | 2.475399153 | -5.513333716 | 3.19E-07 | 1.12E-05 | 6.388559245 |
| P2RY1 | -0.980545801 | 1.742864686 | -8.750379568 | 9.59E-14 | 5.93E-11 | 20.88245398 |
| FIGF | -1.01777726 | 4.117214848 | -5.003592445 | 2.68E-06 | 6.30E-05 | 4.351656175 |
| VIPR1 | -1.03058477 | 3.30883335 | -3.629277843 | 0.000466347 | 0.003815992 | -0.51684919 |
| CCK | -1.037992977 | 1.446205105 | -9.449103579 | 3.24E-15 | 1.19E-11 | 24.15781393 |
| MGAM | -1.050393266 | 1.753835089 | -7.832154875 | 7.96E-12 | 1.71E-09 | 16.6081415 |
| IL18R1 | -1.066830324 | 2.807813205 | -8.729567444 | 1.06E-13 | 6.26E-11 | 20.78504242 |
| FAM107A | -1.097250373 | 3.741610841 | -7.031815933 | 3.49E-10 | 4.01E-08 | 12.95338168 |
| MT1M | -1.107221633 | 3.841933619 | -4.136114788 | 7.79E-05 | 0.000905602 | 1.157675694 |
| EDNRB | -1.118487917 | 2.746984935 | -7.194304627 | 1.63E-10 | 2.04E-08 | 13.68764565 |
| SLC6A4 | -1.123231205 | 2.869403851 | -3.03642214 | 0.003112776 | 0.016920262 | -2.263641944 |
| BTNL9 | -1.133070388 | 2.334835666 | -9.183303038 | 1.18E-14 | 1.53E-11 | 22.9112458 |
| CPB2 | -1.141549315 | 1.801505584 | -3.84918056 | 0.000218387 | 0.002104656 | 0.190471446 |
| HHIP | -1.143900196 | 2.334968309 | -5.869741842 | 6.81E-08 | 3.20E-06 | 7.870362872 |
| NDRG4 | -1.183421263 | 2.404767566 | -8.083934696 | 2.38E-12 | 7.94E-10 | 17.77402617 |
| PLA2G1B | -1.286946392 | 3.472616209 | -4.597849309 | 1.36E-05 | 0.000228831 | 2.810802031 |
| FKBP5 | -1.320509676 | 2.96722243 | -5.969119946 | 4.40E-08 | 2.19E-06 | 8.291001771 |
| CA4 | -1.328367941 | 2.620815789 | -5.163632859 | 1.39E-06 | 3.66E-05 | 4.979807493 |
| ARG1 | -1.336345777 | 1.011896805 | -7.833452422 | 7.91E-12 | 1.71E-09 | 16.61413399 |
| IL18RAP | -1.360956516 | 3.660593085 | -7.225801389 | 1.41E-10 | 1.79E-08 | 13.83049218 |
| SLCO4A1 | -1.442723633 | 0.726064275 | -9.183829416 | 1.17E-14 | 1.53E-11 | 22.91371433 |
| PNMT | -1.464453619 | 1.686357724 | -7.772669721 | 1.06E-11 | 2.14E-09 | 16.33361269 |
| S100A12 | -1.54526289 | 3.607399042 | -5.798840319 | 9.29E-08 | 4.13E-06 | 7.572170861 |
| IL1RL1 | -1.628116477 | 2.773331523 | -6.89488842 | 6.60E-10 | 7.02E-08 | 12.33832807 |
| IL1R2 | -2.110393081 | 3.665202265 | -7.876903126 | 6.43E-12 | 1.52E-09 | 16.81490418 |
