# Supplementary material for: The role of age-related genes in idiopathic pulmonary fibrosis and molecular docking analysis of their drug targets
Source: Front Immunol. 2026 Jan 5;16:1697013. doi: 10.3389/fimmu.2025.1697013 (PMC12812732; doi:10.3389/fimmu.2025.1697013)
Supplement: Supplementary file 5 [file Table3.docx]

**Supplementary table 3:** Age-related differential genes.

| id | logFC | AveExpr | t | P.Value | adj.P.Val | B |
| --- | --- | --- | --- | --- | --- | --- |
| MDK | 1.023878318 | 1.313111962 | 9.731471791 | 8.24E-16 | 5.35E-12 | 25.48141273 |
| PIR | -0.81166719 | 1.331872314 | -9.223215512 | 9.70E-15 | 1.53E-11 | 23.09842613 |
| CBS | -0.672131879 | 1.174610288 | -8.863069076 | 5.56E-14 | 3.80E-11 | 21.41014213 |
| CTH | -0.896025337 | 0.702879697 | -8.462868391 | 3.85E-13 | 1.92E-10 | 19.53834006 |
| IGFBP7 | 0.673751626 | 4.137596215 | 8.00732186 | 3.44E-12 | 9.72E-10 | 17.41864143 |
| TLR4 | -0.691020219 | 2.533189523 | -7.974257204 | 4.03E-12 | 1.05E-09 | 17.26542537 |
| HMGCR | -0.638347371 | 1.789980372 | -7.83221197 | 7.95E-12 | 1.71E-09 | 16.60840518 |
| SOX4 | 0.677517151 | 2.146105254 | 7.798166815 | 9.36E-12 | 1.96E-09 | 16.45123718 |
| MMP7 | 2.042584315 | 5.683870877 | 6.660204274 | 1.95E-09 | 1.77E-07 | 11.29287374 |
| TBX3 | -0.593290796 | 1.35908787 | -6.283291338 | 1.08E-08 | 7.25E-07 | 9.640050727 |
| CLU | 0.693047104 | 2.717056072 | 5.820851187 | 8.44E-08 | 3.82E-06 | 7.66456838 |
| PDZD2 | -0.585313846 | 3.001076002 | -5.464172341 | 3.93E-07 | 1.32E-05 | 6.187675503 |
| LCN2 | 1.478934658 | 4.474537007 | 5.231680146 | 1.05E-06 | 2.91E-05 | 5.250144738 |
| MME | -0.683582016 | 1.977906119 | -5.037209916 | 2.34E-06 | 5.60E-05 | 4.482692138 |
| ALOX15B | -0.911825301 | 3.948187383 | -4.829536053 | 5.42E-06 | 0.000111068 | 3.681251496 |
| GDF15 | 0.636243404 | 1.246956582 | 4.50076525 | 1.97E-05 | 0.000311548 | 2.453899627 |
| AGR2 | 0.777199239 | 2.804165261 | 3.831541435 | 0.000232328 | 0.002213512 | 0.132620976 |
| LY6D | 0.853267579 | 2.137244187 | 3.727954254 | 0.000332944 | 0.002947245 | -0.203221389 |
| SOX2 | 0.886019912 | 0.787282005 | 3.335893792 | 0.001226704 | 0.008221796 | -1.411610022 |
