# Supplementary material for: The role of age-related genes in idiopathic pulmonary fibrosis and molecular docking analysis of their drug targets
Source: Front Immunol. 2026 Jan 5;16:1697013. doi: 10.3389/fimmu.2025.1697013 (PMC12812732; doi:10.3389/fimmu.2025.1697013)
Supplement: Supplementary file 11 [file Table9.docx]

**Supplementary table 9 Molecular docking plots between LCN2 and inulin**

| CurPocket  ID | Vina  score | Cavity  volume | Center  (x, y, z) | Docking size  (x, y, z) |
| --- | --- | --- | --- | --- |
| C1 | -6.5 | 1962 | -12, 19, -21 | 24, 24, 24 |
| C5 | -5.9 | 63 | -1, 3, -9 | 24, 24, 24 |
| C2 | -5.6 | 440 | -7, 1, -19 | 24, 24, 24 |
| C4 | -5.5 | 92 | -17, 26, -3 | 24, 24, 24 |
| C3 | -4.7 | 117 | 2, 8, -23 | 24, 24, 24 |
