# Supplementary material for: The role of age-related genes in idiopathic pulmonary fibrosis and molecular docking analysis of their drug targets
Source: Front Immunol. 2026 Jan 5;16:1697013. doi: 10.3389/fimmu.2025.1697013 (PMC12812732; doi:10.3389/fimmu.2025.1697013)
Supplement: Supplementary file 12 [file Table10.docx]

**Supplementary table 10 Molecular docking plots between Clu and meclizine**

| CurPocket  ID | Vina  score | Cavity  volume | Center  (x, y, z) | Docking size  (x, y, z) |
| --- | --- | --- | --- | --- |
| C1 | -7.7 | 555 | -11, -10, 18 | 23, 23, 23 |
| C3 | -7.2 | 348 | 48, -10, 57 | 23, 23, 23 |
| C2 | -7.1 | 387 | 40, 11, 46 | 23, 23, 23 |
| C4 | -6.9 | 223 | 37, 2, 45 | 23, 23, 23 |
| C5 | -6.5 | 218 | 34, 20, 41 | 23, 23, 23 |
